# Newly Isolated *Streptomyces* sp. JBS5-6 as a Potential Biocontrol Agent to Control Banana Fusarium Wilt: Genome Sequencing and Secondary Metabolite Cluster Profiles

**DOI:** 10.3389/fmicb.2020.602591

**Published:** 2020-12-03

**Authors:** Tao Jing, Dengbo Zhou, Miaoyi Zhang, Tianyan Yun, Dengfeng Qi, Yongzan Wei, Yufeng Chen, Xiaoping Zang, Wei Wang, Jianghui Xie

**Affiliations:** ^1^Haikou Experimental Station, Chinese Academy of Tropical Agricultural Sciences, Haikou, China; ^2^Key Laboratory of Biology and Genetic Resources of Tropical Crops, Ministry of Agriculture, Institute of Tropical Bioscience and Biotechnology, Chinese Academy of Tropical Agricultural Sciences, Haikou, China

**Keywords:** biocontrol, *Streptomyces*, banana fusarium disease, whole genome sequencing, GC-MS

## Abstract

Banana is a key staple food and fruit in countries all over the world. However, the development of the global banana industry is seriously threatened by Fusarium wilt disease, which is caused by *Fusarium oxysporum* f. sp. *cubense* (Foc). In particular, Foc tropical race 4 (Foc TR4) could infect more than 80% of global banana and plantain crops. Until now, there were no commercial chemicals or resistant cultivars available to control the disease. Biological control using actinomycetes is considered a promising strategy. In this study, 88 actinomycetes were isolated from a banana orchard without symptoms of Fusarium wilt disease for more than 10 years. An actinobacterial strain labeled as JBS5-6 has exhibited strong antifungal activities against Foc TR4 and other selected 10 phytopathogenic fungi. Based on phenotypic and biochemical traits as well as complete genome analysis, strain JBS5-6 was assigned to *Streptomyces violaceusniger.* Extracts of the strain inhibited the mycelial growth and spore germination of Foc TR4 by destroying membrane integrity and the ultrastructure of cells. The complete genome of strain JBS5-6 was sequenced and revealed a number of key function gene clusters that contribute to the biosynthesis of active secondary metabolites. Sixteen chemical compounds were further identified by gas chromatography-mass spectrometry (GC-MS). 5-hydroxymethyl-2-furancarboxaldehyde was one of the dominant components in strain JBS5-6 extracts. Moreover, fermentation broth of strain JBS5-6 significantly reduced the disease index of banana seedlings by inhibiting the infection of Foc TR4 in a pot experiment. Hence, strain JBS5-6 is a potential biocontrol agent for the management of disease and the exploitation of biofertilizer.

## Introduction

Banana Fusarium wilt is caused by the soil-borne fungus *Fusarium oxysporum* f. sp. *cubense* (Foc). It is a highly destructive disease that significantly affects the banana industry (Zhou et al., 2019). According to the pathogenicity against banana different cultivars, Foc pathogens can be divided into four physiological tropical races, namely Foc TR1, Foc TR2, Foc TR3, and Foc TR4 (Dita et al., 2010). It is anticipated that Foc TR4 in particular, could infect more than 80% of global banana and plantain crops, which causes the whole plant to wilt and die (Dita et al., 2018). Until now, there has been a lack of effective physical and chemical strategies to manage the disease (Ploetz, 2015). Biological control agents (BCAs) are considered a promising strategy to limit the spread of banana Fusarium wilt. Some microbes such as *Trichoderma* spp., *Pseudomonas* spp. and *Bacillus* spp. have been widely used as BCAs (Harman et al., 2004; Aloo et al., 2019). However, only a few novel antimicrobials have been discovered in recent decades (Sprenger and Fukuda, 2016).

*Streptomyces* belonging to actinomycetes have been studied extensively as potential BCAs against phytopathogenic fungi (Getha and Vikineswary, 2002). Our previous results showed that *Streptomyces* sp. SCA3-4 strongly inhibited spore germination and hyphal development of Foc TR4 in a plate experiment (Qi et al., 2019). *S. violaceusniger* exhibited a 48–52% inhibitory efficiency on banana Fusarium wilt on potted plants (Getha and Vikineswary, 2002). *Streptomyces lunalinharesii* B-03 had a 73% reduction of banana Fusarium wilt disease (Zhou et al., 2017). Similarly, *Streptomyces aureoverticillatus* HN6 and *Streptomyces griseorubiginosus* S96 also exhibited a potential biocontrol for banana Fusarium wilt (Cao et al., 2005; Xing and Wang, 2015). Hence, the isolation of antagonistic actinomycetes is important for controlling banana Fusarium disease.

Accumulated evidence has indicated that *Streptomyces* can produce some well-known antibiotics such as spectinomycin, streptovaricin, and desertomycin, which are the main antimicrobial compounds for protecting plants from pathogens (Kim et al., 2008). Recently, four new antifungal metabolites were discovered from *Streptomyces*, such as (6S,8aS,9S,11S,12aR)-6-hydroxy-9,10-dimethyldecahydrobenzo[d]azecine-2,4,12(3H)-trione (termed as 210-A), fungichromin, 2-methyl-2,5,6-bornantriol,4,4-(3-hydroxypropane-1,1-diyl)diphenol and 7-(4-methoxybenzyl)-4,5,6,7-tetrahydro-1,3-oxazepine-5,6-diol (Wu et al., 2009; Wei et al., 2011). In particular, blasticidin-S from *S. griseochromogenes* was used as an antibiotic commercially in controlling rice blast in Japan (Tapadar and Jha, 2013). However, if this approach is going to be used to manage different phytopathogenic diseases, it is necessary to discover more novel secondary metabolites. Whole-genome sequencing identifies important antimicrobial compounds and it is important to screen high-efficiency secondary metabolites in developing methods for the biocontrol of banana Fusarium disease.

In the present study, a strain of JBS5-6 with a strong antifungal activity was newly isolated from a banana orchard in which there had been no disease symptoms of Foc TR4 for more than 10 years. According to the physiological and biochemical characteristics as well as molecular technology, the strain was identified as *S. violaceusniger.* In addition, strain JBS5-6 also exhibited a wide-spectrum antifungal ability against the selected 10 phytopathogenic fungi. The complete genome of strain JBS5-6 revealed a number of key gene clusters, contributing to the biosynthesis of active secondary metabolites. Gas chromatography-mass spectrometry (GC-MS) was performed to further identify antimicrobial compounds of strain JBS5-6 extracts. In a pot experiment, its fermentation broth significantly improved banana seedling resistance to Foc TR4 by inhibiting the infection of the pathogen.

## Materials and Methods

### Collection of Soil Samples

A banana orchard which had been without symptoms of banana wilt for more than 10 years was selected in Nanbao (109°51′17″E, 19°47′1″N) of Hainan Province, China. An approximate 10–20 cm soil layer of the banana plant rhizosphere was collected in May 2017. Ten individual banana plants with a distance of at least 5 m were randomly selected. Soil samples at four positions of each plant rhizosphere were collected. A total of 40 samples from ten plants were mixed as a soil sample in a sterile plastic bag and stored at −20°C.

### Actinomycetes Isolation

Actinomycetes were isolated by a serial dilution method (Williams et al., 1983). Rifampicin (50 mg/L) and nystatin (50 mg/L) were added to inhibit bacterial and fungal growth. To prepare soil suspension, a 10 g dried soil sample was transferred into a 250-mL bottle. 90 mL of sterile distilled water was added and cultured at 200 rpm for 30 min at 28°C. The soil suspension was then diluted from 10^–3^ to 10^–5^ fold, which were spread on the Gause’s no. 1 medium at 28°C for 7–15 days, respectively. Each colony was purified on the yeast extract-malt extract (ISP2) agar and kept in 20% (v/v) of glycerol at −80°C.

### The Selected Phytopathogenic Fungi

A total of 11 phytopathogenic fungi were selected, including *Fusarium oxysporum* Race 4 (ATCC 76255, Foc TR4) from a banana, *Colletotrichum acutatum* (ATCC56815) from loquat, *Curvularia fallax* (ATCC 38579) from banana, *Fusarium oxysporum* sp. *cucumebrium* (ACCC30220) from cucumber, *Pyricularia oryzae* (ATCC 52352) from rice, *Colletotrichum gloeosporioides* (ATCC 58222) from mango, *Fusarium graminearum* (ATCC 46779) from wheat, *Botryosphaeria dothidea* (ATCC 208829) from apple, *Curvularia lunata* (ATCC 42011) from maize, *Colletotrichum fragariae* (ATCC 58718) from strawberry and *Botrytis cinerea* (ATCC 11542) from grape. These fungi were kindly provided by the Institute of Environment and Plant Protection at the China Academy of Tropical Agricultural Sciences, Haikou, China.

### Antifungal Activity Assay

Antagonistic actinomycetes were screened according to the inhibition ability against Foc TR4 using a conventional spot inoculation method (Qi et al., 2019). A phytopathogenic fungal disk (8.5 mm diameter) was placed in the center of the potato dextrose agar (PDA) plate. Four mycelial blocks (5 mm diameter) of each actinomycete were inoculated at four symmetrical points about 2.5 cm from the plate center (Yun et al., 2018). A fungal disk alone was used as a control. After incubation at 28°C for 5–7 days, the inhibition zone was recorded by measuring the distance between the fungal mycelium edge and the actinomycete disks. The inhibition percentage of the radial growth (PIRG) was calculated using the following formula: PIRG = [(C – T)/C] × 100, where C and T represented the radius of fungal mycelial growth in the control and the treatment groups, respectively. All strains were tested in triplicate experiments.

### Morphological Characteristics of Actinomycetes

The morphological and cultural characteristics of actinomycetes were assayed according to the protocol of the International Streptomyces Project (ISP) (Shirling and Gottlieb, 1966). The strain was cultured in different culture media (ISP2, ISP3, ISP4, ISP5, ISP6, ISP7, and PDA) at 28°C for 7–10 days. The colors of aerial mycelia and diffusible pigments were determined in comparison with the ISCC-NBS color charts (Kelly, 1958). The morphological characteristics of actinomycetes including growth, aerial mycelium, spore, and hypha were observed after 14 days using a scanning electron microscopy (SEM, Zeiss Sigma VP, Germany).

### Physiological and Biochemical Characteristics of Actinomycetes

The effects of different pH, temperature, and NaCl on microbial growth were measured on the ISP4 medium after incubation at 28°C for 14 days. The pH range was set from 3.0 and 11.0 at intervals of one unit. The temperature ranged from 4, 15, 20, 25, 28, 37, 40, and 45°C. NaCl tolerance for microbial growth was tested in the presence of 0–15% at intervals of one unit. Some biochemical characteristics, including H_2_S production, milk peptonization, starch hydrolysis, nitrate reduction, tyrosinase production, and cellulose hydrolysis, were also measured according to the description of Qi et al. (2019). The carbon and nitrogen sources were tested according to the previous method of Chen et al. (2018).

### DNA Extraction and PCR Amplification

The genomic DNA of actinomycetes was extracted using a rapid genomic DNA extraction kit (DP1301, Beijing BioTek Biotech Co., Ltd., China). The 16S *rRNA* gene was amplified using a pair of conserved primers (27F, 5′-AGAGTTTGATCMTGGCTCAG-3′) and (1492R, 5′-TACGGYTACCTTGTTACGACT-3′) (Gupta et al., 2014). A 50-mL reaction system contained 1 μL of 27F primer (10 mmol/L), 1 μL of 1492R primer (10 mmol/L), 25 μL of 2 × Taq Master Mix, and 2 μL of genomic DNA. PCR was performed in the professional Trio PCR System (Biometra, Göttingen, Germany). The PCR reaction system included denaturation at 95°C for 3 min, followed by 38 cycles (94°C for 30 s, 56°C for 1 min, and 72°C for 2 min), and a final extension at 72°C for 10 min. The amplified products were visualized by 1.0% of agarose gel electrophoresis and were sequenced by the Huada Gene Technology Co., Ltd (Shenzhen, China).

### Construction of Phylogenetic Tree

The amplified 16S *rRNA* sequence was compared with those that have been deposited in public databases and the EzBiocloud server^1^ (Yoon et al., 2017). The similarity of pairwise sequences was also calculated. Other 16S *rRNA* sequences from representative taxa were obtained from GenBank databases using the CLUSTAL X software (Thompson et al., 1997). A phylogenetic tree was constructed using the neighbor-joining method of MEGA version 7.0 (Kumar et al., 2016). The evolutionary distance was estimated by bootstrap analysis.

### Antifungal Activity of *Streptomyces* Extracts

The selected *Streptomyces* were inoculated in one liter of sterilized soybean liquid culture medium (SLM, 15 g of corn flour, 10 g of glucose, 0.5 g of K_2_HPO_4_, 0.5 g of NaCl, 0.5 g of MgSO_4_, 3 g of beef extract, 10 g of yeast extract, 10 g of soluble starch, 2 g of CaCO_3_, pH 7.2–7.4) at 180 rpm for 7 days at 28°C (Hong et al., 2009). The culture filtrate was extracted with EtOAc at a ratio of 1:1 (v/v). The mixture was filtered through the qualitative filter paper (Whatman no. 1). The organic phase (EtOAc) was separated from the filtered liquid media using a decantation funnel. The extracts were evaporated using a rotary vacuum evaporator (EYELA, N-1300, Japan), and then dissolved in 10% of dimethyl sulfoxide (DMSO) with a final concentration of 20.0 mg/mL. After being sterilized by filtration through a 0.22-μm sterile filter (Millipore, Bedford, MA, United States), the extracts were stored at −4°C.

### Effect of *Streptomyces* Extracts on Growth Inhibition of Fungal Mycelia

A total of 11 phytopathogenic fungi were selected for analyzing the antifungal activities of *Streptomyces* extracts using a plate method (Sharma et al., 2016). The extracts were dissolved in 10% of DMSO (20.0 mg/mL) and added to the PDA plate with a final concentration of 200 μg/mL. An equivalent 10% of DMSO was used as a control. A fungal disk (5 mm diameter) was inoculated aseptically into the center of each Petri dish at 28°C. When the control mycelium reached the edge of the plate, the diameter of the mycelium was measured. Each assay was performed in three replicates. The inhibition percentage of mycelial growth was calculated using the following formula: mycelial inhibition percentage = [(C – T)/C] × 100, where C and T represented the colonial diameters of control and different treatments. 50% of the growth inhibition of pathogenic mycelia were defined as an effective concentration (EC_50_) of extracts.

### Determination of the Minimum Inhibitory Concentration (MIC) of *Streptomyces* on Phytopathogenic Fungi

The minimum inhibitory concentrations (MICs) of *Streptomyces* extracts on different phytopathogenic fungi were determined by a method of 96-well microtiter assay (Chen et al., 2018). Cycloheximide and Nystatin were used as positive controls. 10% of DMSO was used as a negative control. The different concentrations (50.0, 25.0, 12.5, 6.25, 3.125, 1.563, and 0.781 μg/mL) were prepared for MIC tests. Each well contained 80 μL of the Roswell Park Memorial Institute (RPMI) mycological media, 100 μL of fungal suspension with 1.0 × 10^5^ CFU (colony-forming units)/mL, and 20 μL of *Streptomyces* extracts. The 96-well plates (Nunc MicroWell, Roskilde, Denmark) were covered with a plastic lid and incubated at 28°C for 12 h. Absorbance was measured at 620 nm using a microplate photometer (Packard Spectra Count, Packard Instrument Co., Downers Grove, IL, United States). The lowest concentration of growth inhibition was recorded as MIC.

### Effects of *Streptomyces* Extracts on the Spore Germination of Phytopathogenic Fungi

The inhibitory activities of *Streptomyces* extracts on spore germination of phytopathogenic fungi were observed using light microscopy (Nikon, E200MV, Japan). Each fungal strain was challenged in dose response to different extract concentrations (1/2 × EC_50_, 1 × EC_50_, or 2 × EC_50_). The mixture (0.1 mL) of extracts and fungal spore suspension (10^5^ CFU/mL) at a ratio of 1:1 (v/v) was placed on a sterile glass slide and incubated in a moist chamber at 28°C for 20–24 h. For the control, we replaced extracts with sterile water. All experiments were performed in three replicates. We detected approximately 100 conidia in each field. The percentage of spore germination (PSG) was calculated as follows: PSG = (A – B)/A, where A and B represented the spore germination rate of control and treatment groups, respectively (Chen et al., 2018).

### Effects of *Streptomyces* Extracts on the Hyphae Growth of Foc TR4

The hyphal growth of Foc TR4 treated with *Streptomyces* extracts was observed according to the method of Feng et al. (2019). A mycelial disk (4 mm diameter) was taken from the periphery of a colony growing on the PDA medium with 2 × EC_50_ of extracts and incubated at 28°C for 24 h. 10% of DMSO treatment was used as a negative control. Foc TR4 hyphae were chosen from the plate margin, fixed in 2.5% of glutaraldehyde (0.1 mol/L of phosphate buffer, pH 7.2) at 4°C overnight, and post-fixed in 1% of OsO_4_ for 2 h at 4°C. The samples were then dehydrated in a graded ethanol series, dried, and coated with gold powder. Hyphal growth was observed using SEM at an accelerating voltage of 3 kV.

### Effects of *Streptomyces* Extracts on the Ultrastructure of Foc TR4

A 5-mm diameter mycelial disk was removed from the periphery of a colony that has been growing on the PDA medium for 24–72 h at 28°C. An equal volume of DMSO (10%, v/v) was used as a control. The sample was pre-fixed in 3% of fresh glutaraldehyde, post-fixed in 1% of osmium tetroxide solution, and dehydrated by transferring fixed specimens to a graded water-acetone series (50, 70, 80, 90, 95, and 100% for 10 min, respectively). Afterward, these samples were embedded in Epon 812 resin at 37°C for 12 h, 45°C for 12 h, and 60°C for 24 h, respectively. The embedded materials were sectioned with an ultramicrotome (EM UC6, Leica, Germany). These sections were double stained with saturated uranyl acetate and lead citrate and observed by a transmission electron microscopy (TEM, HT7700, Hitachi, Japan) under an operating voltage of 80 kV.

### Effects of *Streptomyces* Extracts on the Physiological and Biochemical Characteristics of Foc TR4

Spore suspension of Foc TR4 with 10^7^CFU/mL was cultured in 100 mL of the PDB medium at 180 rpm for 48 h at 28°C. Different concentrations (0, 5, 10, 25, 50, 100, and 200 μg/mL) of extracts were added and co-cultured at 180 rpm for 72 h at 28°C, respectively. Foc TR4 mycelia were collected by centrifugation at 5,000 rpm for 20 min and then washed three times with sterile water. One gram of Foc TR4 mycelia was ground in 5 mL of Tris–HCl on the ice. After centrifugation at 10,000 rpm for 20 min at 4°C, the supernatant was used for measuring N-acetylglucosamine content in the light of the standard curve (Hofmann and Roitsch, 2000). Two grams of mycelia were ground in 25 mL of sodium acetate (0.05 mol/L, pH 5.0). After centrifugation at 12,000 rpm for 15 min at 4°C, crude enzyme supernatant was used for measuring the activity of β-1,3-glucanase according to the method of Miller (1959). One unit of β-1,3-glucanase activity was defined as an enzyme amount that produced 1 μmoL of reducing sugar per min. Soluble protein content was measured in the mixture (0.1 mL of supernatant, 0.9 mL of distilled water, and 5 mL of Coomassie brilliant blue G-250 dye). The absorbance was determined at 595 nm and protein content was calculated by comparing the standard curve (Bradford, 1976). The dried mycelia of Foc TR4 were weighed after treatment with different extract concentrations, respectively.

### Genome Sequencing and Metabolite Prediction of the Selected *Streptomyces*

*Streptomyces* were cultured aerobically in the ISP4 medium at 28°C for 48–72 h. Genomic DNA was extracted using the Wizard^®^ Genomic DNA Purification Kit (Promega, Madison, WI, United States) according to the standard manufacturer’s protocol. Purified genomic DNA was quantified by a TBS-380 fluorometer (Turner BioSystems Inc., Sunnyvale, CA, United States). Sequencing and assembly of the complete genome were performed by the Majorbio Bio-Pharm Technology Co., Ltd., Shanghai, China. The data generated from the Illumina platform were used for bioinformatics analysis using the I-Sanger Cloud Platform^2^. The protein-coding genes were predicted by Glimmer v3.02 (Delcher et al., 2007). Gene functions were annotated by the Prokaryotic Genome Annotation Pipeline of NCBI (Tatusova et al., 2016). The biosynthetic gene clusters of secondary metabolites were predicted using the online antiSMASH v4.2.0 software (Huang et al., 2019). Analysis of average nucleotide identity (ANI) was aligned using the online OrthoANI (Yoon et al., 2017). The GC content was calculated according to its complete genome sequence.

### GC-MS Analysis

Gas chromatography-mass spectrometry was used to identify the chemical compounds in *Streptomyces* extracts according to a previous method with a slight modification (Krishnamoorthy et al., 2018). Briefly, GC-MS was performed on a Shimadzu GC 2010 plus with a triple quadrupole mass spectrometer (TP-8030), which was fitted with a DB-5 ms (5% phenyl methylsiloxane) capillary column of dimensions 30 m × 0.25 mm × 0.25 μm. Helium as a carrier gas was injected at 1 mL/min. The column temperature was programmed initially at 60°C for 1 min, increased to 100°C at 5°C/min for 5 min, raised at 10°C/min to 250°C for 35 min, and finally kept to 280°C at 10°C/min for 25 min. The mass spectrometer was operated in electron ionization (EI) mode at 70 eV, with an interface temperature of 280°C, an ion source temperature of 240°C, a mass spectrometer acquisition delay time of 3.5 min, and a continuous scan from 50 to 650 amu. Peaks were identified in comparison with the mass spectra data against the National Institute of the Standards and Technology (NIST) spectral library (Wang et al., 2015).

### Pot Experiment of Banana Seedlings

A pot experiment was performed to test the inhibition ability of the selected *Streptomyces* against Foc TR4 between May and July 2017. The soil was sampled from the Nanbao banana orchard, and passed through a twenty-mesh sieve, and sterilized at 160°C for 2 h. 900 g of soil was added to each plastic pot. Banana seedlings (Foc TR4-susceptible Cavendish cultivar “Brazilian”) were washed with sterile water and planted in plastic pots. The Foc TR4 fungi constitutively expressing a *GFP* gene was provided by the Chinese Academy of Tropical Agricultural Sciences Environment and Plant Protection Institute, Haikou, China. Fungal spore suspension of GFP-tagged Foc TR4 was inoculated into the soil at the concentration of 2 × 10^5^CFU/g soil. Meanwhile, the selected *Streptomyces* was cultured in sterilized soybean liquid culture medium (SLM) at 150 rpm for 7 days at 28°C. The fermentation broth was filtered through two layers of sterile prewetted Mira cloth and was diluted to 50 folds. 100 mL of fermentation broth was added to the roots of each banana seedling. The treated banana seedlings were transferred into a greenhouse at 30°C, 60% humidity for 12 h dark/12 h light. Each experiment was repeated in triplicate and 10 plants were used in each repeat. Three groups were set including CK1 (inoculated with Foc TR4), CK2 (inoculated with sterile water), and treatment (inoculated with Foc TR4 and a fermentation broth of *Streptomyces*).

### Assay of Biocontrol Efficiency

After co-incubation for 35 days, the corms of the banana seedlings were cut to detect the infection degree of Foc TR4. Root sections were made using the hand-sliced method. The infection and colonization of Foc TR4 were observed using a laser scanning confocal microscope (ZEISS, LSM800, Germany) equipped with filter blocks with spectral properties matching those of GFP (488 nm excitation, 520∼540 nm long-pass emission). The disease indexes of banana seedlings were measured after 35 d according to the following formula (Fang, 2007):

Disease⁢Index=Σ(Numberofdiseasedplantsofeachgrade×valueofrelativegrade)/(Totalnumberinspected×4)×100

The number of chlorotic leaves was used to evaluate the disease grade such as a healthy plant (grade 0), below 25% of chlorotic leaves (grade 1), 25–50% of chlorotic leaves (grade 2), 51–75% of chlorotic leaves (grade 3), and 76–100% of chlorotic leaves (grade 4).

Biocontrol efficiency was measured using the following formula:

Controllingefficiency(%)=(diseaseindexinCK1-diseaseindexinthetreatedgroup)/diseaseindexinCK1×100

## Results

### Actinomycetes Isolation and Antifungal Activity Assay

**FIGURE 1:**
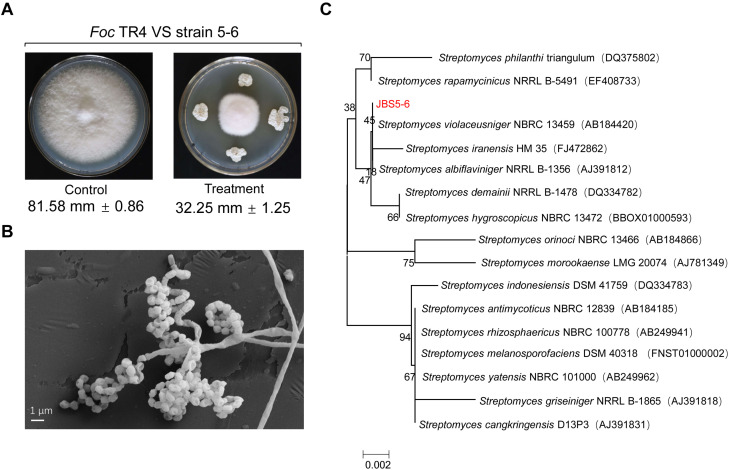
Isolation and identification of a *Streptomyces* strain with high antifungal activity against Foc TR4. **(A)** A newly isolated strain labeled with JBS5-6 exhibited high antifungal activity against Foc TR4. **(B)** Morphological characteristics of aerial mycelia and spores of strain JBS5-6 using SEM. **(C)** A phylogenetic tree was constructed based on 16S *rRNA* sequences using the neighbor-joining method. Bootstrap percentages at nodes were calculated with 1,000 replicates. GenBank accession numbers were listed in brackets. Bar, 0.002 substitutions per nucleotide position.

A total of 88 actinomycetes were isolated from the banana orchard without symptoms of banana wilt disease for more than 10 years. All isolates were screened against Foc TR4. Out of them, 17 actinomycetes showed antifungal activities during the preliminary experiment. In particular, a strain marked with JBS5-6 exhibited strong antifungal activity against Foc TR4 with the inhibition efficiency of 60.46% (Figure 1A and Supplementary Table 1).

### Cultural and Morphological Characteristics of Strain JBS5-6

Strain JBS5-6 can grow well on ISP3, ISP4, ISP5, and ISP6 media with pH 5.0-9.0 and less than 3% of NaCl at 28–45°C (Supplementary Tables 2, 3). The strain could resolve nitrate and hydrolyze hippurate, but could not produce soluble pigment, H_2_S, and tyrosinase. Moreover, it also cannot hydrolyze starch, solidify milk, decompose cellulose, and liquefy gelatin (Supplementary Table 3). SEM analysis showed that the straight and long aerial mycelia produced about 4–6 spores at the end of the short branch. Smooth and cylindrical spores first formed spiral chains, and then gathered together like a bunch of grapes (Figure 1B).

### Phylogenetic Construction of Strain JBS5-6

The complete 16S *rRNA* gene (1509 bp) of strain JBS5-6 was amplified and was submitted to the GenBank database with the accession number MN384963. By analysis of EzBioCloud, the strain showed a 99.93% similarity with the standard strain *S. violaceusniger* NBRC13459. A phylogenetic tree was constructed using the neighbor-joining method of MEGA version 7.0. These selected strains could be divided into four major clusters (Figure 1C). Strain JBS5-6 formed a well-delineated subclade with *S. violaceusniger* and the bootstrap value was over 45. Combining the morphological, physiological, and biochemical characteristics, strain JBS5-6 was assigned to the genus *Streptomyces*.

### Carbon and Nitrogen Utilization of Strain JBS5-6

Strain JBS5-6 could fully utilize 17 carbon sources including α-lactose, D-cellobiose D-fructose, D-galactose, D-glucose D-mannose, D-sorbitol, D-trehalose, D-xylose, melibiose, D-mannitol, inositol, melezitose, rhamnose, ribose, maltose, and sucrose (Supplementary Table 4). However, it could not use L-arabinose and melitose as carbon sources. Furthermore, the stain could fully utilize L-serine, glycine, and histidine as nitrogen resources, but could not utilize L-arginine, L-phenylalanine, methionine, tryptophan, (+)-cysteine, phenylalanine, valine, and glutamate (Supplementary Table 4).

### Antifungal Activity Assay of Strain JBS5-6

To assess whether strain JBS5-6 had a broad-spectrum antifungal activity, the 11 phytopathogenic fungi were selected (Supplementary Table 5). The percentage of inhibition of mycelial growth ranged from 70.37 to 88.89%. Compared with untreated hypha, more than 80% of inhibitory abilities were demonstrated against Foc TR4 (ATCC 76255), *C. acutatum* (ATCC 56815), *C. gloeosporioides* (ATCC 58222), and *C. lunata* (ATCC 42011). The maximum inhibition percentage of mycelial growth was detected against *C. lunata* (ATCC 42011) (88.98 ± 1.47%), followed by Foc TR4 (ATCC 76255) (87.22 ± 1.47%). The minimum inhibition percentage was observed against *F. oxysporum. sp. cucumebrium* (ACCC 30220) (72.22 ± 0.56%), followed by *P. oryzae* (ATCC 52352) (69.44 ± 2.42) and *B. dothidea* (ATCC 208829) (70.37 ± 2.31). Based on the acquired toxicity curves, EC_50_ values of strain JBS5-6 extracts were measured against 11 pathogenic fungi (Supplementary Table 5). Its extracts exhibited the best antifungal activity against *C. gloeosporioides*, *C. lunata*, *B. cinerea*, and Foc TR4 with an EC_50_ value of 106.64, 108.96, 129.27, and 136.92 μg/mL, respectively. These results indicate that strain JBS5-6 could have a potentially excellent inhibitory ability against the selected 11 pathogenic fungi.

### Strain JBS5-6 Extracts Significantly Inhibit Spore Germination and Hyphal Growth of Foc TR4

**FIGURE 2:**
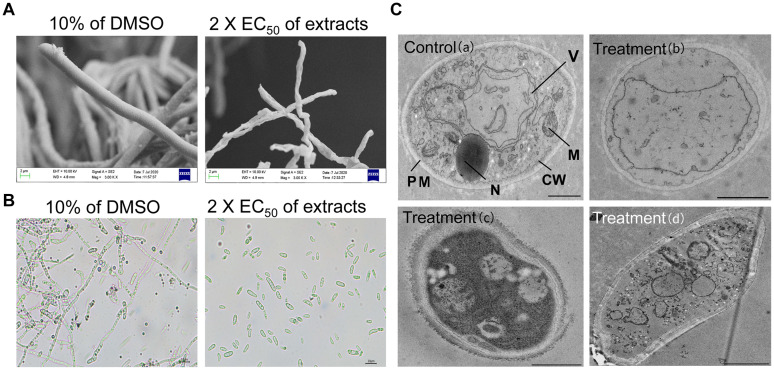
Effects of strain JBS5-6 extracts on hyphal morphology **(A)**, spore germination **(B)**, and cell ultrastructure of Foc TR4 **(C)**. A treatment with 10% DMSO was used a control. Scale bars represent 1 μm in **(a,b)**, 0.5 μm in **(c)**, and 2 μm in **(d)**. CW, cell wall; PM, plasma membrane; M, mitochondrion; N, nucleus; V, vacuole.

The morphological characteristics of Foc TR4 hypha after treatment with strain JBS5-6 extracts were detected by SEM (Figure 2A). In the control PDA plate with 10% of DMSO, Foc TR4 mycelia displayed smooth external surfaces, rounded apexes, and regular morphology. Most of the formed fungal mycelia appeared to be parallel and adherent. In the treatment group, Foc TR4 mycelia exhibited a deformed and wrinkled external surface and a varied hyphal diameter. The mycelia showed an undulation and deformation structure along with the hyphal borders. Some ruptured bubbles at the top of mycelia were also detected. Similarly, strain JBS5-6 extracts also inhibited significantly the hyphal growth of the selected phytopathogenic fungi (Supplementary Table 5).

Additionally, strain JBS5-6 extracts were used to analyze their effects on spore germination of the tested pathogenic fungi (Figure 2B). The results showed that spore germination was significantly reduced (*P* < 0.05) after treatment with different concentration extracts. At 2 × EC_50_, strain JBS5-6 extracts effectively inhibited the conidial germination of Foc TR4, *C. acutatum*, *C. gloeosporioides*, and *C. lunata* with the inhibition percentages of 80.81, 83.25, 82.23, and 82.38%, respectively (Supplementary Table 5). The percentage of spore germination gradually decreased with the increase of concentration of extracts. The minimum inhibition percentage of spore germination was 64.31 ± 0.98% against *P. oryzae* (ATCC 52352), followed by 65.13 ± 0.38% against *B. dothidea* (ATCC 208829). By contrast, 10% of DMSO (v/v) treatment did not inhibit the spore germination of the tested pathogenic fungi.

### Effects of Strain JBS5-6 Extracts on the Ultrastructure of Foc TR4 Cells

Ultrastructure of hyphae cells treated with 2 × EC_50_ of strain JBS5-6 extracts was observed by TEM. In the 10% of the DMSO treatment group, cell wall, membrane, and organelles were intact and well defined. Organelles such as vesicles, vacuoles, lipid bodies, and mitochondria were observed clearly (Figure 2C). After treated with strain JBS5-6 extracts, the cell wall of Foc TR4 mycelia became incrassated with a rough surface. Hyphal cells showed more low density granules and less high density granules. In addition, the cytoplasmic heterogeneity and disintegrated organelles were also observed in the treated cells (Figure 2C).

### Effects of Strain JBS5-6 Extracts on the Physiological and Biochemical Characteristics of Foc TR4

**FIGURE 3:**
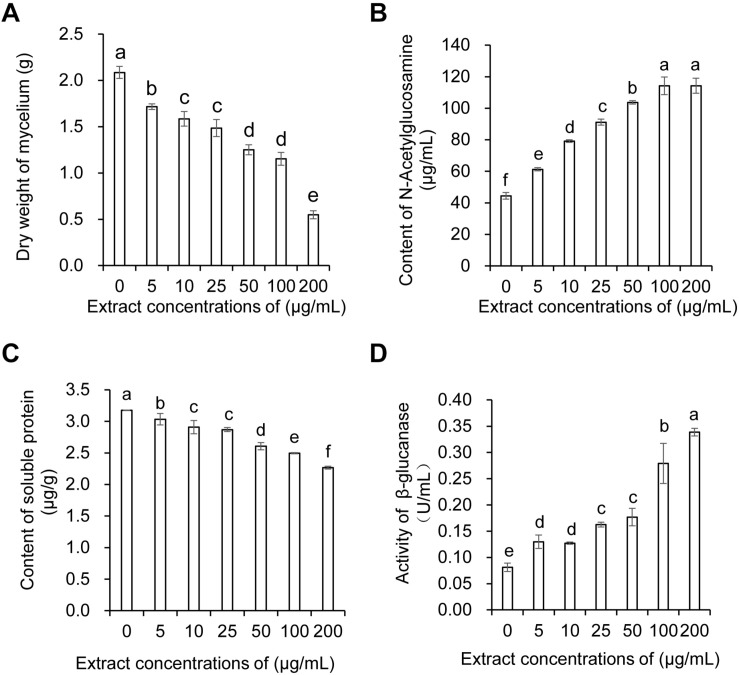
Effect of strain JBS5-6 extracts on dry weight of mycelia **(A)**, N-Acetylglucosamine contents **(B)**, soluble protein contents, and **(C)** β-glucanase activity **(D)** of Foc TR4. All experiments were repeated in triplicates. Different lower-case letters indicated a significant difference at the *P* < 0.05 level by Duncan’s new multiple range test.

The dry weight of Foc TR4 mycelia gradually decreased with the concentration increase of strain JBS5-6 extracts. No significant difference was observed between the 10 and 25 μg/mL treatment groups. Although 50 and 100 μg/mL extracts significantly inhibited the increase of dry weight of Foc TR4 mycelia, there was no obvious difference between them (Figure 3A). Compared with the control group, the soluble protein contents of Foc TR4 mycelia exhibited a decreasing tendency with the increase of extract concentrations (Figure 3C). No significant difference in soluble protein content was detected between 10 and 25 μg/mL extract treatments. The lowest soluble protein content was observed in 200 μg/mL of the treatment group. In addition, N-acetylglucosamine and β-glucanase activities of Foc TR4 significantly increased along with the increase of extract concentrations, suggesting that the cell wall of Foc TR4 was seriously damaged by strain JBS5-6 extracts (Figures 3B,D).

### Minimum Inhibitory Concentration of Strain JBS5-6

The MICs of strain JBS5-6 extracts were determined against 11 phytopathogenic fungi by the 96-well microtiter assay. Compared with the antifungal activity of cycloheximide and azoxystrobin, MICs ranged from 50 μg/mL to 1.563 μg/mL. The low MICs were detected against Foc TR4 with 6.25 μg/mL and *F. graminearum* with 3.125 μg/mL. The lowest MIC was 1.563 μg/mL against *C. gloeosporioides*, suggesting that extracts had a strong fungicidal activity that inhibits the growth of this pathogen (Supplementary Table 6). However, 10% of DMSO had no inhibitory effect on the selected phytopathogenic fungi in the control group.

### Genome Sequencing and Metabolite Prediction of Strain JBS5-6

**FIGURE 4:**
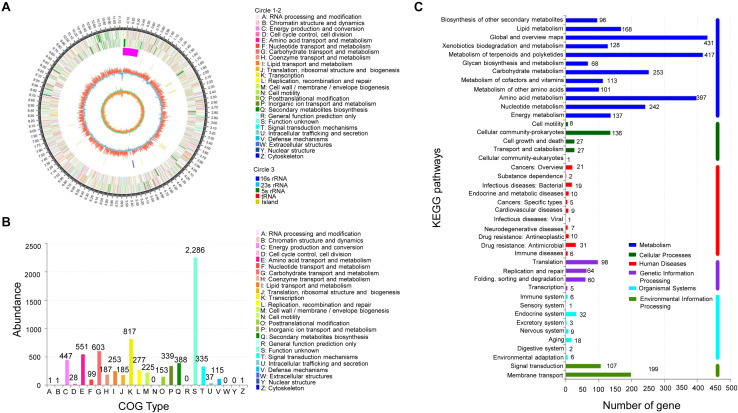
Analysis of genome structure and metabolic pathway of strain JBS5-6. **(A)** Circular map of strain JBS5-6 chromosome. From outside to center, rings 1 and 4 show protein-coding genes colored by COG categories on forward/reverse strand. Rings 2 and 3 showed CDS, tRNA, and rRNA on forward/reverse strand. Ring 5 represented the G + C content, followed by G + C skew in ring 6. **(B)** COG annotation of strain JBS5-6 genome. **(C)** Pathway annotation of strain JBS5-6 genome according to the KEGG database. The vertical axis represented the level two classification of KEGG pathway. The horizontal axis represented the gene number annotated in this classification. Different colors of the columns represented different classifications of KEGG pathway.

By sequencing and assembly, the genome of strain JBS5-6 consisted of 11,161,721 bp with 71.40% of GC content, including 9840 genes and 73 rRNA genes (Figure 4A and Supplementary Table 7). Furthermore, an ANI calculated for genomes of strain JBS5-6 and *S. violaceusniger* NBRC 13459^T^ was 97.52, which was above the threshold value of 95–96% for species delineation (Richter and Rosselló-Móra, 2009). Strain JBS5-6 was identified as *S. violaceusniger.* Among a total of identified 9767 protein-coding genes, 73.85% and 45.38% of them were annotated into COG and KEGG functional categories, respectively (Figures 4B,C). For COG categories, the highest ratio was the metabolism process (39.12%), followed by information storage and processing (17.48%) as well as cellular processes and signaling (12.20%). Notably, 31.20% of the high proportion showed in the poorly characterized category. Sixty-five biosynthetic gene clusters coding for secondary metabolites were detected in strain JBS5-6 genome, mainly including 23 type I PKS gene clusters, one type II PKS gene cluster, two type III PKS gene clusters, ten NRPS gene clusters, six terpene gene clusters, two arylpolyene-ladder gene clusters, three bacteriocin gene clusters, two butyrolactone gene clusters, one ectoine gene cluster, one hserlactone gene cluster, one indole cluster, six lantipeptide gene clusters, one oligosaccharide cluster, three siderophore gene clusters, and other gene clusters (Supplementary Table 8).

By alignment of antiSMASH software and GenBank, above 70% of similarity gene clusters producing twenty chemicals were annotated in Supplementary Table 9, including 12 gene clusters with 100% similarity. The structures and functions of predicted chemicals were demonstrated (Supplementary Figure 2). Genome analysis further revealed that nine gene clusters were involved in the biosynthesis of antimicrobial metabolites, including azalomycin F3a, amipurimycin, curamycin, halstoctacosanolide A, myxothiazol, echoside A-E, bicornutin A1-A2, icosalide A-B, and nigericin. In addition, two gene clusters may participate in the biosynthesis of anticancer agents (nigericin and azalomycin F3a). One gene cluster was responsible for the production of cytotoxic agents (rhizomide A-C and luminmide). Furthermore, two gene clusters were involved in the biosynthesis of desferrioxamine B and E, which were used as the iron chelation removing excess iron in the environment. Notably, other unmatched gene clusters were also found in the strain JBS5-6 genome, which might participate in the biosynthesis of some key secondary metabolites (Supplementary Table 9).

### Identification of the Active Compound Candidates of Strain JBS5-6 by GC-MS

The active compounds of strain JBS5-6 extracts were analyzed by GC-MS. A total of 16 chemical compounds were identified by alignment of the NIST library based on retention time, molecular mass, and the molecular formula (Table 1). These compounds mainly contained hydrocarbons, flavonoids, polymeric aldehyde, acids, pyrrolizidine, and phenol esters. Their chemical structures and functions were predicted in Supplementary Figure 1, including: (1): 1,3-dimethyl-benzene; (2): 4H-Pyran-4-one,2,3-dihydro-3,5-dihydroxy-6-methyl-; (3): 5-hydroxymethyl-2-furancarboxaldehyde; (4): Benzeneacetic acid; (5): Methyl 1-methylpyrrole-2-carboxylate; (6): 5-Acetyl-2-furanmethanol; (7): N-Methylpyrrole-2-carboxylic acid; (8): phenylethyl ester of caffeic acid; (9): 1H-Indene,2,3-dihydro-1,1,3-trimethyl-3-phenyl-; (10): 4-methyl-N-(tetrahydro-2-oxo-3-furanyl)-Benzenesulfonamide; (11): 2,5-Piperazinedione,3,6-bis(2-methylpropyl)-; (12): Phenol,2,2’-methylenebis[6-(1,1-dimethylethyl)-4-methyl-; (13): Pyrrolo[1,2-a]pyrazine-1,4-dione,hexahydro-3-(phenylmethyl)-; (14): 2-Cyclohexen-1-one, 3-methyl-4,4-diphenyl-; (15): n-Hexadecanoic acid and (16): Pyrrolo[1,2-a]pyrazine-1,4-dione,hexahydro-3-(2-methylpropyl)-. The peak area of compounds represented the proportion to their quantities in extracts (Table 1). Among these compounds, the peak area of 5-hydroxymethyl-2-furancarboxaldehyde was 52.75%, indicating that it was the dominant compound in strain JBS5-6 extracts.

**TABLE 1:** Compounds identified from the crude extract of strain JBS5-6 through GC-MS.

No.	Predicted compounds	RT (min)	Similarity (%)	Area (%)	MF	MM	Activity	References
1	1,3-dimethyl-benzene	4.55	29.67	1.49	C_8_H_10_	106	Organic intermediate	Liu et al., 2007
2	4H-Pyran-4-one, 2,3-dihydro-3,5-dihydroxy-6-methyl	12.00	96.23	2.78	C_6_H_8_O_4_	144	Antioxidant and antifungal activity	Teoh and Don, 2014
3	5-hydroxymethyl-2-furancarboxaldehyde	16.39	95.99	52.75	C_6_H_6_O_3_	126	An intermediate of agricultural chemicals	Muratore et al., 2006
4	Benzeneacetic acid	16.54	31.24	0.68	C_8_H_8_O_2_	136	Antimicrobial activity	Valsalam et al., 2019
5	Methyl 1-methylpyrrole-2-carboxylate	17.35	66.70	1.08	C_7_H_9_NO_2_	139	No activity reported	
6	5-Acetyl-2-furanmethanol	17.77	13.89	0.34	C_7_H_8_O_3_	140	No activity reported	
7	N-Methylpyrrole-2-carboxylic acid	19.94	58.01	1.99	C_6_H_7_NO_2_	125	No activity reported	
8	Phenethyl ester of caffeic acid	23.06	39.14	0.60	C_17_H_16_O_4_	284	Antifungal activity	Pittalà et al., 2018
9	1H-Indene, 2,3-dihydro-1,1,3-trimethyl-3-phenyl	29.01	17.58	0.97	C_18_H_2_0	236	No activity reported	
10	4-methyl-N-(tetrahydro-2-oxo-3-furanyl)-Benzenesulfonamide	29.16	8.69	0.91	C_11_H_13_NO_4_S	255	No activity reported	
11	2,5-Piperazinedione, 3,6-bis(2-methylpropyl)	29.26	65.08	1.04	C_12_H_22_N_2_O_2_	226	No activity reported	
12	Phenol, 2,2′-methylenebis[6-(1,1-dimethylethyl)-4-methyl	31.73	96.49	5.05	C_23_H_32_O_2_	340	No activity reported	
13	Pyrrolo[1,2-a] pyrazine-1,4-dione,hexahydro-3(phenylmethyl)	31.86	88.36	0.78	C_14_H_16_N_2_O_2_	244	Antimicrobial activity	Melo et al., 2014
14	2-Cyclohexen-1-one, 3-methyl-4,4-diphenyl	35.7	34.57	0.21	C_19_H_18_O	262	No activity reported	
15	Pyrrolo[1,2-a]pyrazine-1,4-dione,hexahydro-3-(2-methylpropyl)	26.81	55.32	0.92	C_11_H_18_N_2_O_2_	210	Antimicrobial activity	Melo et al., 2014
16	n-Hexadecanoic acid	26.53	71.52	8.95	C_16_H_32_O_2_	256	Cytotoxic, Anti-Inflammatory Property	Johannes et al., 2016

### Fermentation Broth of Strain JBS5-6 and Reducing the Disease Index of Banana Seedlings

**FIGURE 5:**
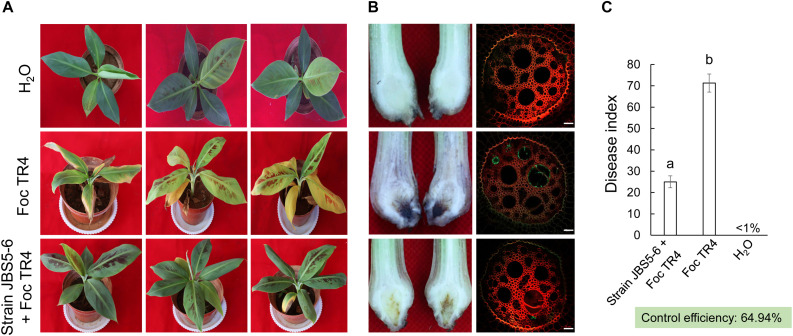
Fermentation broth of strain JBS5-6 improving the resistance of banana seedlings to Foc TR4. **(A)** Chlorotic morphology of leaves inoculated with strain JBS5-6 and Foc TR4 after 35 days. **(B)** Infection characteristics of Foc TR4 in the corms and roots of banana seedlings after 35 days. **(C)** Quantitative analysis of disease index of banana seedlings after 35 days. All experiments were repeated in triplicates. Different lower-case letters indicated a significant difference at the *P* < 0.05 level by Duncan’s new multiple range test.

In our pot experiments, the visual external wilt symptoms (chlorotic leaves) of banana seedlings in the CK1 group (plantlets inoculated with Foc only) was first observed on the seventh day. After that, leaf chlorosis began to transfer from old leaves to young leaves. On day 35, fusarium wilt disease spread rapidly to the whole plant. The leaves with chlorotic symptoms collapsed and a large area of banana corm gradually decayed (Figure 5A). The disease index in the CK1 group reached 71.30 ± 7.24. Although few plantlets in the treatment group of fermentation broth of strain JBS5-6 showed chlorotic symptom on the margin of lower leaves, vascular discoloration was not observed on cutting suckers horizontally (Figure 5A). Only a slight brown in the middle of the banana corm was detected. The disease index was 25.78 and the control efficiency was 64.94% (Figure 5C). The laser scanning confocal microscope was used to future monitor the characteristics of Foc TR4 infection. In the control group, the vascular tissue was infected by Foc TR4 seriously and a number of vascular cells were full of GFP-tagged Foc TR4 spores. However, only a few vascular cells with the GFP-tagged Foc TR4 spores were observed in the treatment group (Figure 5B). These results indicated that the fermentation broth of strain JBS5-6 could inhibit the infection of Foc TR4 and reduce the disease index of banana seedlings. Based on these findings, strain JBS5-6 could improve the resistance of banana seedlings to Foc TR4.

## Discussion

Plant diseases caused by phytopathogenic fungi can cause heavy losses for agriculture production. Banana is an important food crop in the tropics and subtropics, and fusarium wilt is one of the most destructive diseases affecting agricultural production of this fruit. Biological control is a promising strategy for combating fusarium wilt, due to the advantages of high efficiency, broad spectrum, and the fact that it is relatively environmentally friendly (Zhou et al., 2013; Wei et al., 2020). Previous studies have indicated that specific microbial groups, such as *Pseudomonas*, *Streptomyces*, *Flavobacterium*, etc., are closely related to plant disease suppression (Mendes et al., 2011; Cha et al., 2016; Kwak et al., 2018). Similarly, we also found that some functional microbes existed in the selected banana orchard without symptoms of banana wilt for more than 10 years (Zhou et al., 2019). *Streptomyces* and its natural antibiotics were beneficial for constructing a stable disease-suppressive soil microbial community and reducing the incidence index of banana wilt disease (Cha et al., 2016). In our present study, strain JBS5-6 was isolated from the disease-suppressive soil in the above banana orchard. Strain JBS5-6 exhibited a strong antifungal ability against Foc TR4 and the selected 10 different phytopathogenic fungi. Similar studies have also shown that *Streptomyces* exhibit great potential in suppressing plant diseases caused specifically by fungal pathogens (Qin et al., 2017; Chen et al., 2018).

Accumulated evidence indicates that *Streptomyces* species can provide a rich source of natural products that may have potential BCAs (Newman and Cragg, 2016). Most secondary metabolites are used as antibiotics, antitumor agents, antioxidants, pesticides, and plant-growth-promoting substances, etc, (Qi et al., 2019). In our study, strain JBS5-6 extracts exhibited a strong inhibition ability on mycelial growth and the cellular integrity of phytopathogenic fungi (Figure 2 and Supplementary Table 5). Similarly, morphological abnormalities in *F. oxysporum* fungal hyphae were observed after treatment with supernatant of *Streptomyces bikiniensis* HD-087 (Zhao et al., 2014). It was supported by the significant increase of N-Acetylglucosamine content and β-glucanase activity in the treated Foc TR4 mycelial cells (Figure 3). *Streptomyces* sp. M4 treatment caused swelling of fungal mycelia (Sharma et al., 2016). We did not find swollen mycelia in Foc TR4 treated with strain JBS5-6 extracts. This might be because the swollen morphology was an earlier symptom of abnormalities (Wei et al., 2020). The antifungal ability of *Streptomyces* might be related to antibiosis, nutrient competition, degradative enzyme biosynthesis, nitrous oxide production, and quorum quenching (Cohen and Mazzola, 2006). However, the antifungal mechanism of strain JBS5-6 on Foc TR4 still needs to be further investigated.

Additionally, actinomycetes can produce a wide range of bioactive secondary metabolites. Approximately two-thirds of available antibiotics have been isolated from actinomycetes (Wang et al., 2015). The genome sequencing of actinomycetes has also revealed numerous cryptic secondary metabolite gene clusters of unknown and known functions (Figure 4). These gene clusters are responsible for the production of microbial natural products. Biosynthetic gene cluster encoding for polyketides and non-ribosomal peptides were identified in strain JBS5-6 genome, mainly including 23 type I PKS gene clusters, one type III PKS gene cluster, two type III PKS gene clusters, and 10 NRPS gene clusters. The encoding of the genes PKS-I and NRPS might play a role in the production of antifungal activity from strain JBS5-6. Passari et al. (2015) and Sharma et al. (2016) also showed that actinomycetes possessing antifungal activity are positively related to two biosynthetic pathways.

In order to further uncover some important chemicals produced by strain JBS5-6, some gene clusters that encode antibiotic metabolites were identified (Supplementary Figure 2). These include the amipurimycin gene cluster of strain JBS5-6, which showed 90% similarity with the corresponding genes in *S. novoguineensis*. Amipurimycin is a kind of peptidyl nucleoside antibiotic and a potential new source of antimicrobials (Serpi et al., 2016). Yuan et al. (2013) obtained seven azalomycin F analogs (1–7) from a broth of mangrove *Streptomyces* sp. 211726, which were used successfully against *Candida albicans, Staphylococcus aureus, Fusarium moniliforme*, and *F. oxysporum* (Arai, 1968; Chandra and Nair, 1995; Xu et al., 2018). Additionally, one gene cluster of strain JBS5-6 had 83% similarity with the gene involved in the production of nigericin. The compound belonged to a fungistatic polyether with a high inhibitory effect for mycelial growth of *Pythium* and *Phytophthora* (Myskiw et al., 2010). Interestingly, three gene clusters in strain JBS5-6 genome shared 100, 80, and 90% of similarity with desferrioxamine E, desferrioxamine B, and coelichelin, respectively. They were involved in the production of siderophores. Recent research has provided evidence that the secreted siderophores could suppress the pathogen *in vitro* and protect plants from pathogen infection by changing rhizosphere microbiome members (Gu et al., 2020). Hence, strain JBS5-6 might produce some important secondary metabolites to improve its antifungal activity.

To further identify antifungal compounds, GC-MS was used to analyze the secondary metabolites of strain JBS5-6 extracts in our study. A total of 16 chemical compounds were detected, including hydrocarbons, flavonoid, polymeric aldehyde, acids, pyrrolizidine, and phenol esters (Table 1 and Supplementary Figure 1). Of these, 4H-pyran-4-one, 2,3-dihydro-3,5-dihydroxy-6-methyl-(DDMP) isolated from *Schizophyllum commune* received much attention for its antifungal activity against rubberwood fungi (Teoh and Don, 2014). Recent reports showed that benzeneacetic acid exhibited a high inhibition activity against *Pseudomonas aeruginosa* and *Penicillium notatum* (Valsalam et al., 2019). Caffeic acid phenethyl ester was identified as having multiple biological activities, including antioxidant, antimicrobial, anti-inflammatory, and antitumor effects (Pittalà et al., 2018). Hexadecanoic acid exhibited an antibacterial activity by damaging the cell walls of phytopathogenic fungi (El Mokni et al., 2019). Interestingly, these compounds identified by GC-MS were different from those synthesized by the above gene clusters (Table 1 and Supplementary Figure 2). This might be due to the diffidence of identification methods and alignment databases (Wei et al., 2020). Thus, we propose that these compounds could altogether contribute to the broad-spectrum antifungal activity of stain JBS5-6 against the tested fungal pathogens. Compared with the pot experiment, strain JBS5-6 also demonstrated good control efficiency for banana Fusarium disease with a 64.94% inhibition percentage. The above results suggest that in the future the strain will be exploited as a potential biological control agent for pathogenic fungi.

### Conclusion

In this study, *S. violaceusniger* JBS5-6 with strong antifungal activity against Foc TR4 were isolated and identified from the soil of a banana orchard. Its extracts inhibited the mycelial growth and spore germination of Foc TR4. Some key function gene clusters related to the biosynthesis of active secondary metabolites were found in the sequenced complete genome of strain JBS5-6. In addition, 16 chemical compounds were identified by GC-MS. These gene clusters and compounds altogether contribute to the broad-spectrum antifungal activity of stain JBS5-6. This result was supported by the pot experiment, as part of which a fermentation broth of strain JBS5-6 significantly improved the resistance of banana seedlings to Foc TR4.

## Data Availability Statement

The datasets presented in this study can be found in online repositories. The names of the repository/repositories and accession number(s) can be found below: https://www.ncbi.nlm.nih.gov/genbank/, SAMN15515113 https://www.ncbi.nlm.nih.gov/genbank/, MN384963.

## Author Contributions

TJ, DZ, XZ, WW, and JX developed the ideas and designed the experiment. DZ, JX, and WW supervised the research and provided funding support. TJ, DZ, MZ, TY, DQ, YW, and XZ performed the experiments. TJ, DZ, TY, DQ, YW, XZ, WW, and JX analyzed the data. TJ, DZ, WW, and JX prepared the manuscript. All authors contributed to the article and approved the submitted version.

## Conflict of Interest

The authors declare that the research was conducted in the absence of any commercial or financial relationships that could be construed as a potential conflict of interest.

## Footnotes

1 https://www.ezbiocloud.net/identify

2 http://www.i-sanger.com/

## Supplementary Material

Supplementary Figure 1 Chemical structures of the identified compounds of strain JBS5-6 extracts using GC-MS.

Supplementary Figure 2 Genome-wide analysis of gene clusters related to the biosynthesis of secondary metabolites using the online antiSMASH v4.2.0 software.

Supplementary Table 1 Inhibitory activities of 17 actinomycetes isolates on mycelial growth of Foc TR4.

Supplementary Table 2 Growth characteristics of strain JBS5-6 on different solid culture media.

Supplementary Table 3 Physiological and biochemical characteristics of strain JBS5-6.

Supplementary Table 4 Carbon and nitrogen utilization characteristics of strain JBS5-6.

Supplementary Table 5 Inhibitory activities of strain JBS5-6 extracts on mycelial growth and spore germination of the tested phytopathogenic fungi.

Supplementary Table 6 MIC values of strain JBS5-6 extracts against the tested phytopathogenic fungi.

Supplementary Table 7 Summary of strain JBS5-6 genome.

Supplementary Table 8 Cluster number and gene number shown in different cluster types.

Supplementary Table 9 Prediction and functional annotation of secondary metabolites.
