# Supplementary material for: Newly Isolated Streptomyces sp. JBS5-6 as a Potential Biocontrol Agent to Control Banana Fusarium Wilt: Genome Sequencing and Secondary Metabolite Cluster Profiles
Source: Front Microbiol. 2020 Dec 3;11:602591. doi: 10.3389/fmicb.2020.602591 (PMC7744762; doi:10.3389/fmicb.2020.602591)
Supplement: Supplementary Figure 1 — Chemical structures of the identified compounds of strain JBS5-6 extracts using GC-MS. [file Data_Sheet_1.doc]

**Newly Isolated *Streptomyces* sp. JBS5-6 as a Potential Biocontrol Agent to Control Banana Fusarium Wilt: Genome Sequencing and Secondary Metabolite Cluster Profiles**

(Tao Jing, Dengbo Zhou, Miaoyi Zhang, Tianyan Yun, Dengfeng Qi, Yongzan Wei, Yufeng Chen, Xiaoping Zang, Wei Wang, Jianghui Xie)


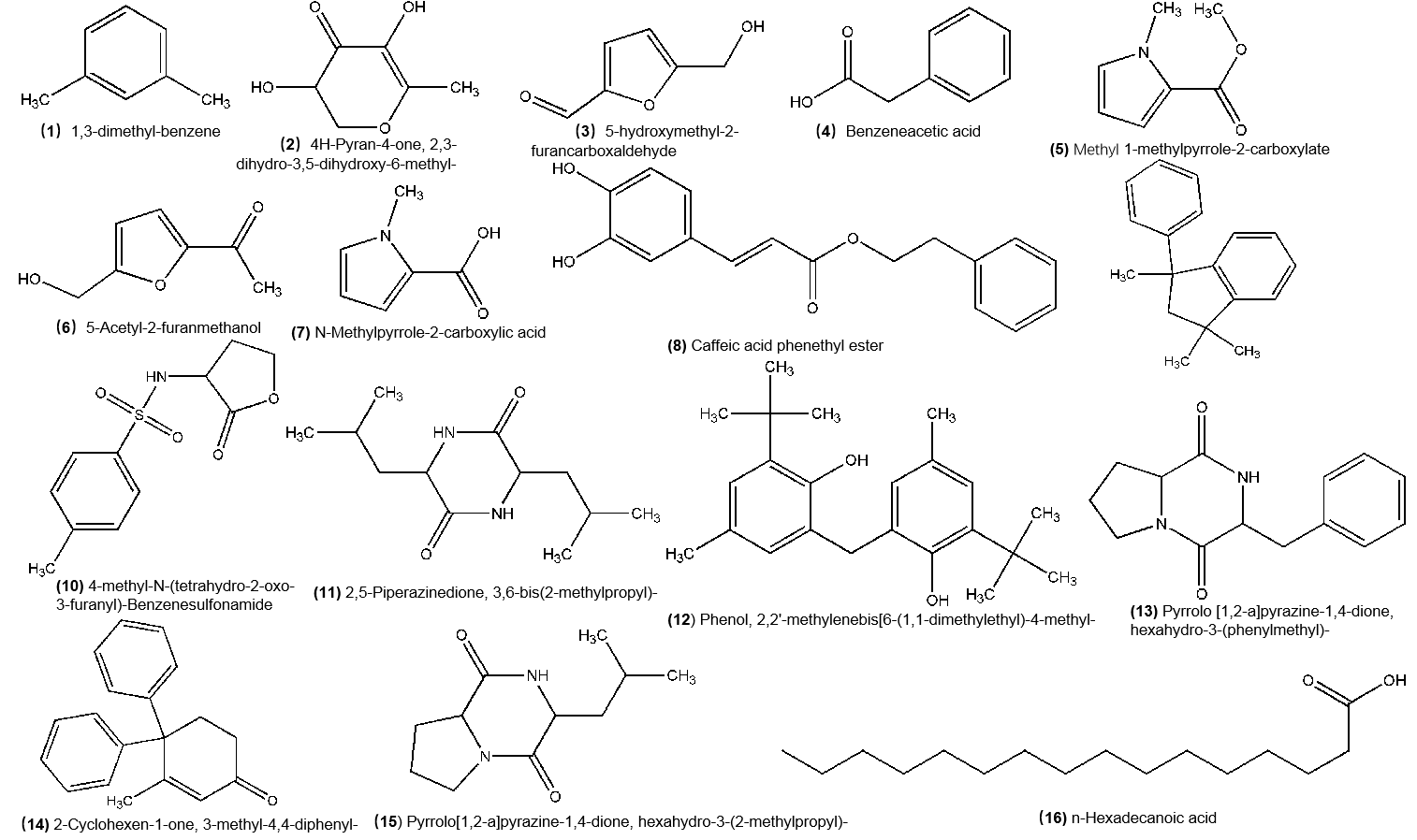


**Figure S1.** Chemical structures of the identiﬁed compounds of strain JBS5-6 extracts using GC-MS.


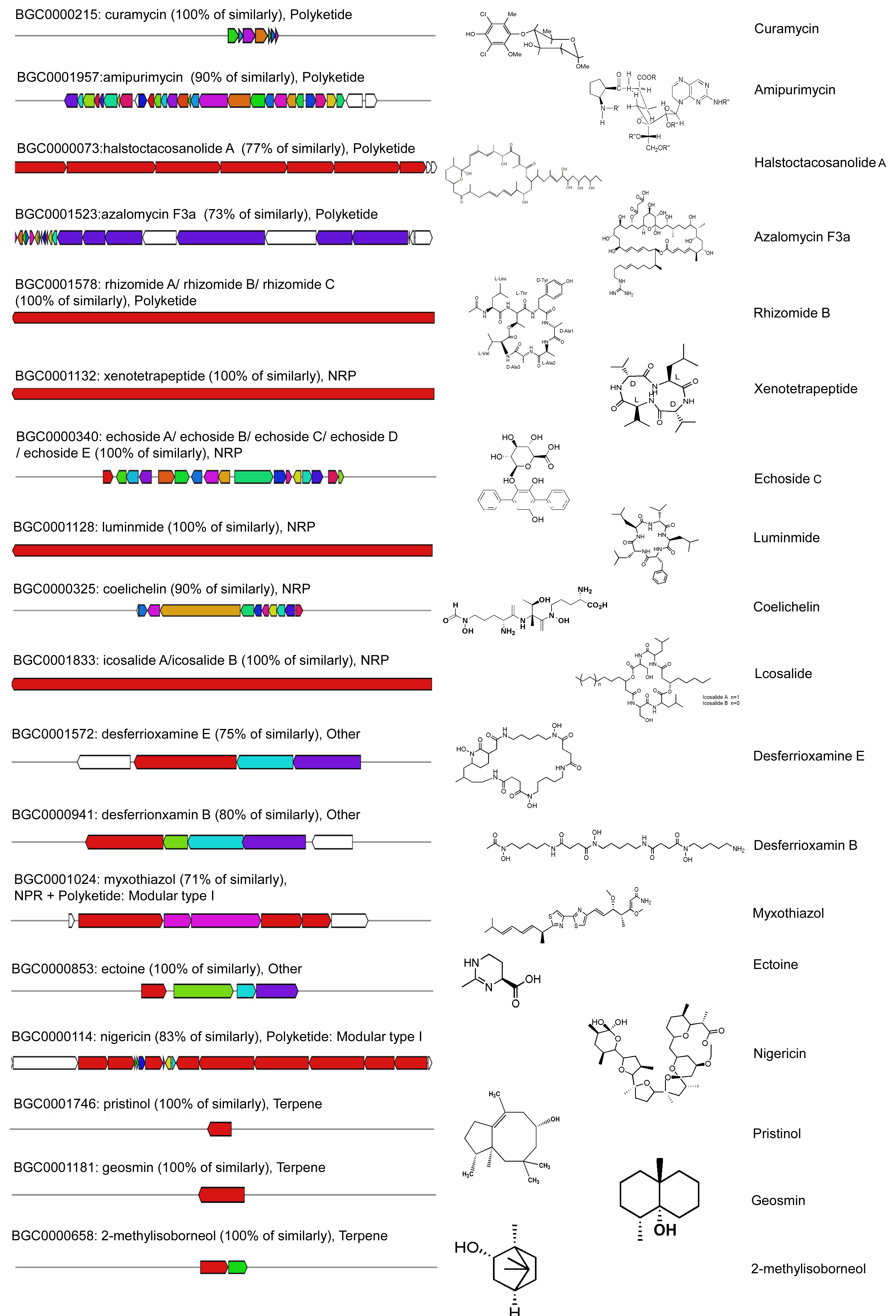


**Figure S2** Genome-wide analysis of gene clusters related to the biosynthesis of secondary metabolites using the online antiSMASH v4.2.0 software.

**Table S1** Inhibitory activities of 17 actinomycetes isolates on [mycelial](../../../../D:/360%25E6%259D%2580%25E6%25AF%2592/Youdao/Dict/8.9.2.0/resultui/html/index.html#/javascript:;) [growth](../../../../D:/360%25E6%259D%2580%25E6%25AF%2592/Youdao/Dict/8.9.2.0/resultui/html/index.html#/javascript:;) of Foc TR4

| **Strain** | **Colony diameters (mm)** | **Mycelial Inhibition (%)** |
| --- | --- | --- |
| JBS1-2 | 42.49 ± 0.96 def | 47.91 ± 1.05 def |
| JBS2-4 | 36.9 ± 1.27 gh | 54.77 ± 1.57 bc |
| JBS2-3 | 54.43 ± 2.81 c | 33.28 ± 3.45 g |
| JBS3-4 | 54.58 ± 1.03 c | 33.09 ± 1.39 g |
| JBS3-1 | 46.01 ± 0.97 d | 43.61 ± 1.86 f |
| JBS3-2 | 35.86 ± 1.05 hi | 56.05 ± 3.30 ab |
| JBS5-6 | 32.25 ± 1.25 i | 60.46 ±1.40 a |
| JBS5-7 | 44.73 ± 2.16 d | 45.17 ± 2.65 f |
| JBS10 | 60.59 ± 0.97 b | 25.73 ± 1.20 h |
| JBS11 | 54.39 ± 2.76 c | 33.33 ± 3.38 g |
| JBS12 | 38.84 ± 1.30 fgh | 52.39 ± 1.59 bcd |
| JBS15 | 60.13 ± 1.98 b | 26.3 ± 2.43 h |
| JBS45 | 36.83 ± 1.78 gh | 54.85 ± 2.19 bc |
| JBS71 | 43.32 ± 1.03 de | 46.89 ± 1.26 ef |
| JBS75 | 40.21± 1.44 efg | 50.71± 1.76 cde |
| JBS77 | 69.29± 0.72 a | 15.06± 0.88 i |
| JBS81 | 51.20± 1.49 c | 37.24± 1.83 g |

Data in the table are means ± SD. Different capital letters in the same column were signiﬁcantly different at the level of P < 0.01 by Duncan’s new multiple range test.

**Table S2**. Growth characteristics of strain JBS5-6 on different solid culture media

| **Medium** | **Aerial mycelium** | **Vegetative mycelium** | **Soluble pigment** | **Growth** | **Colony** 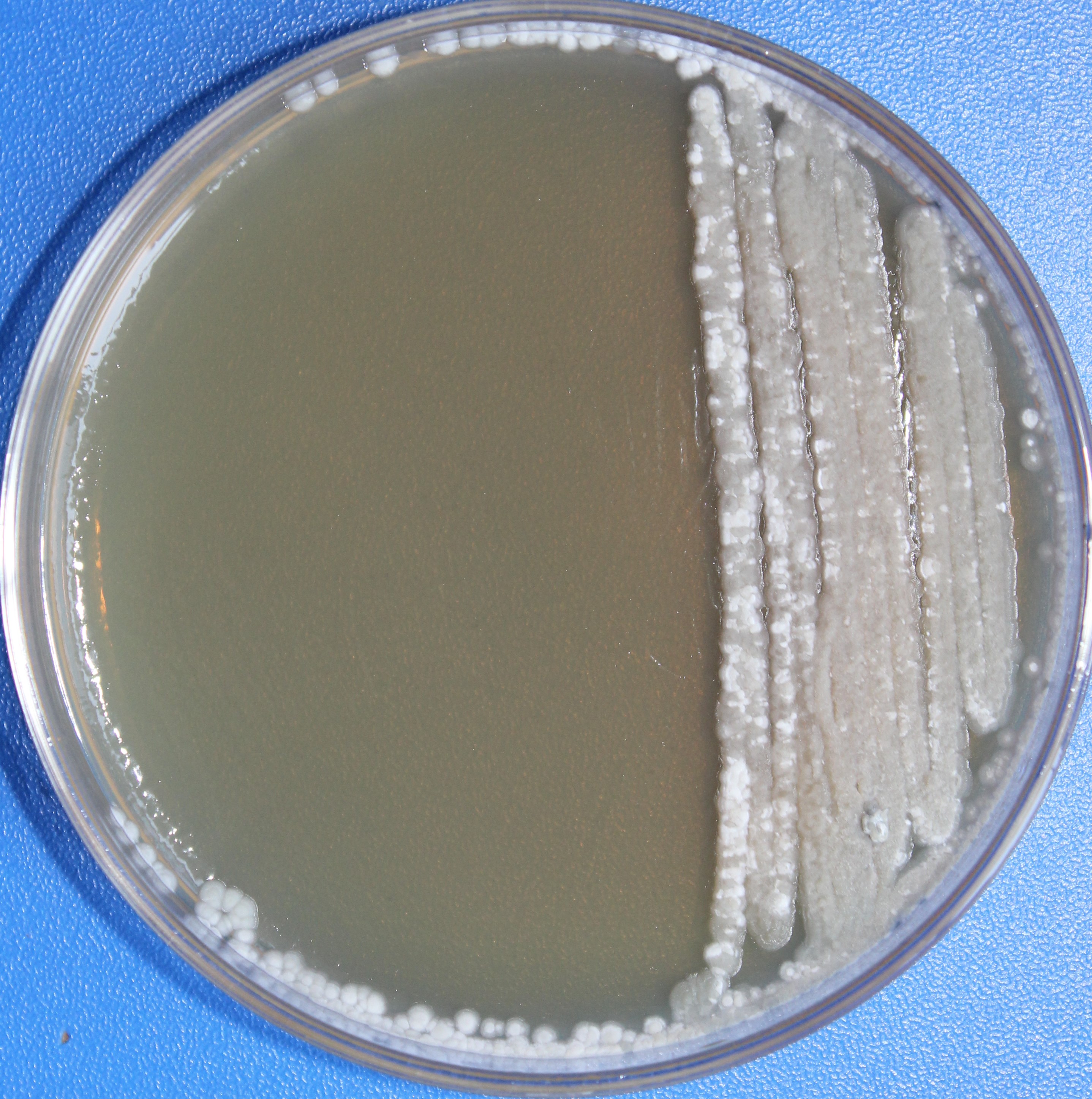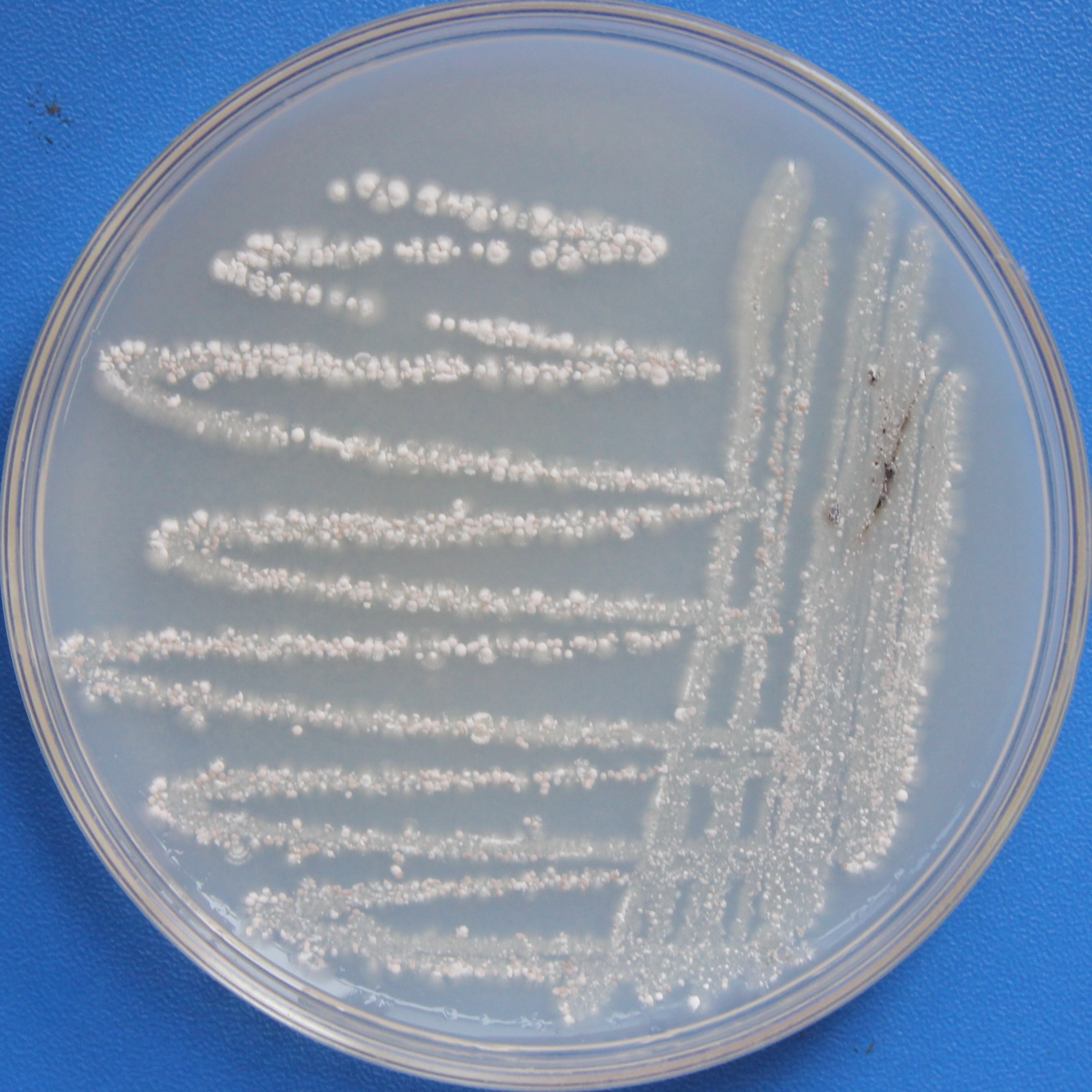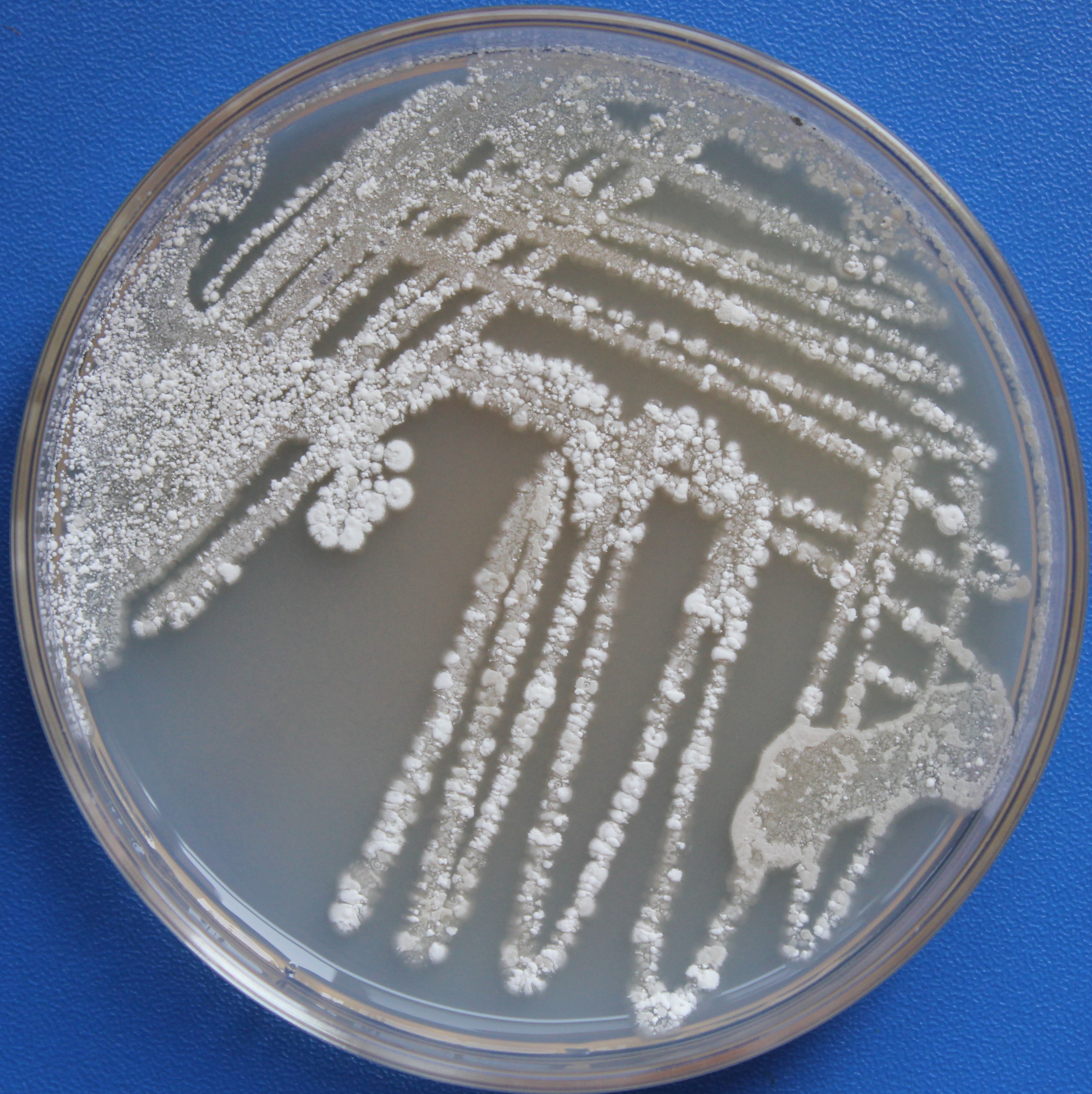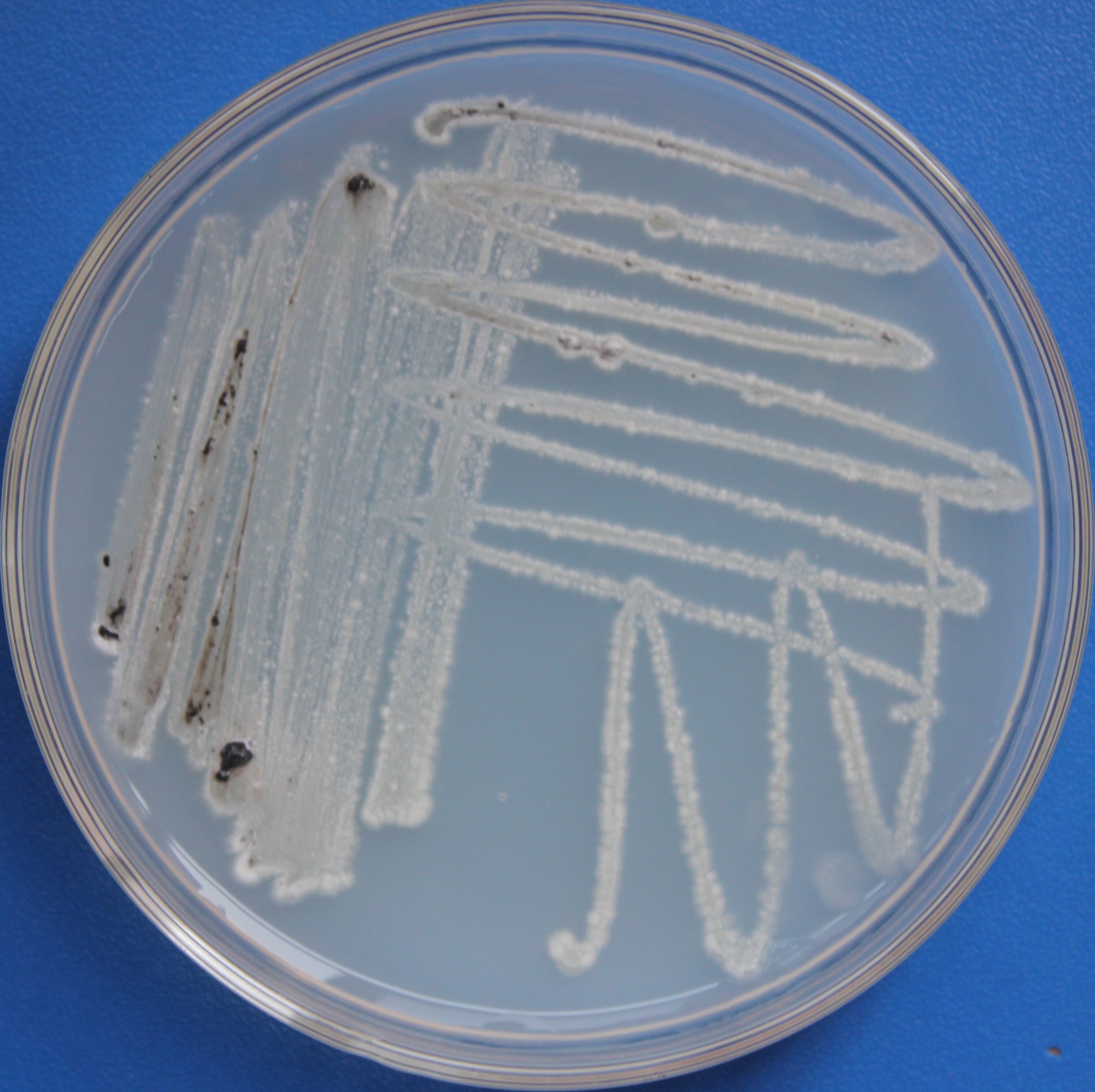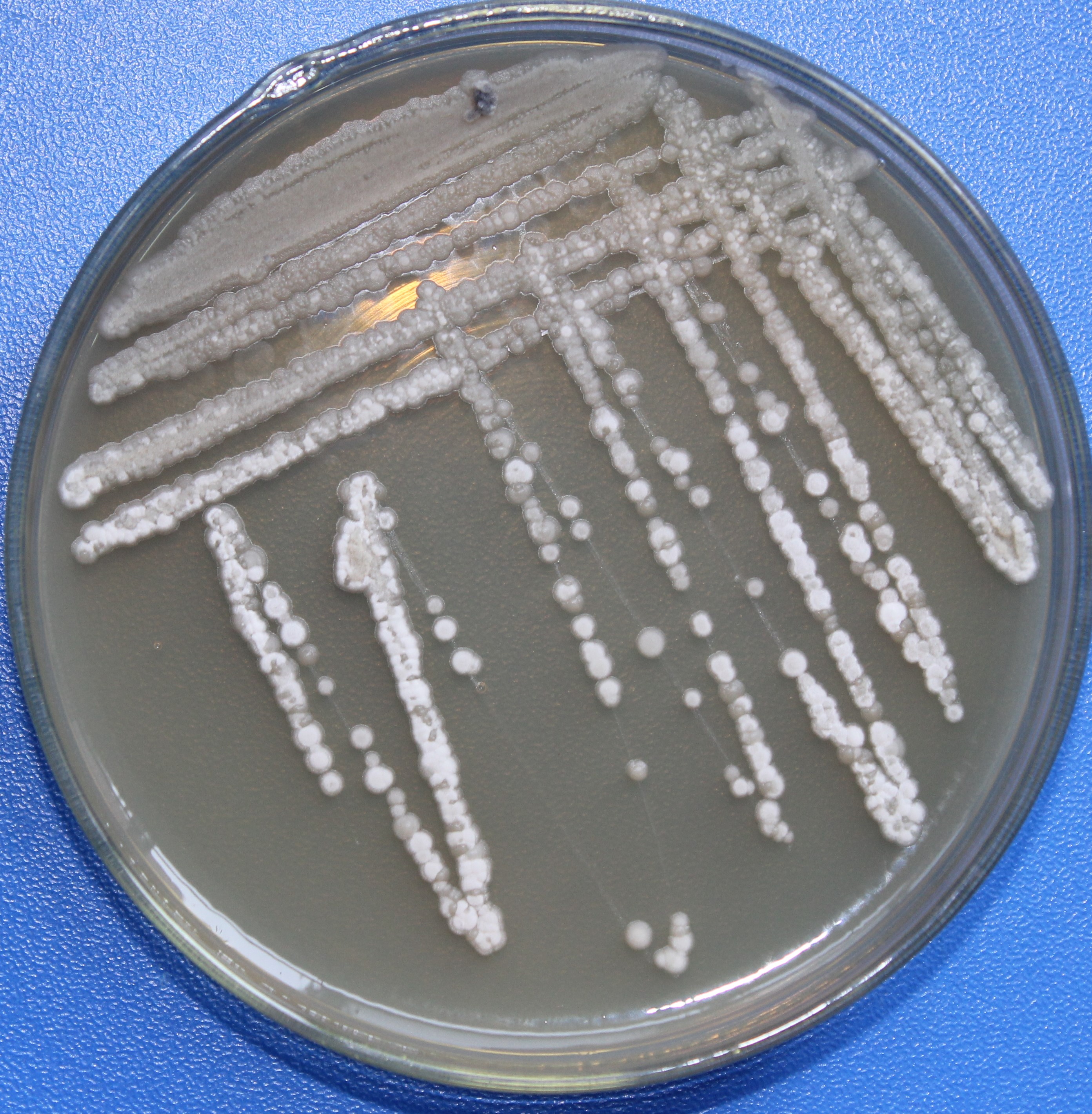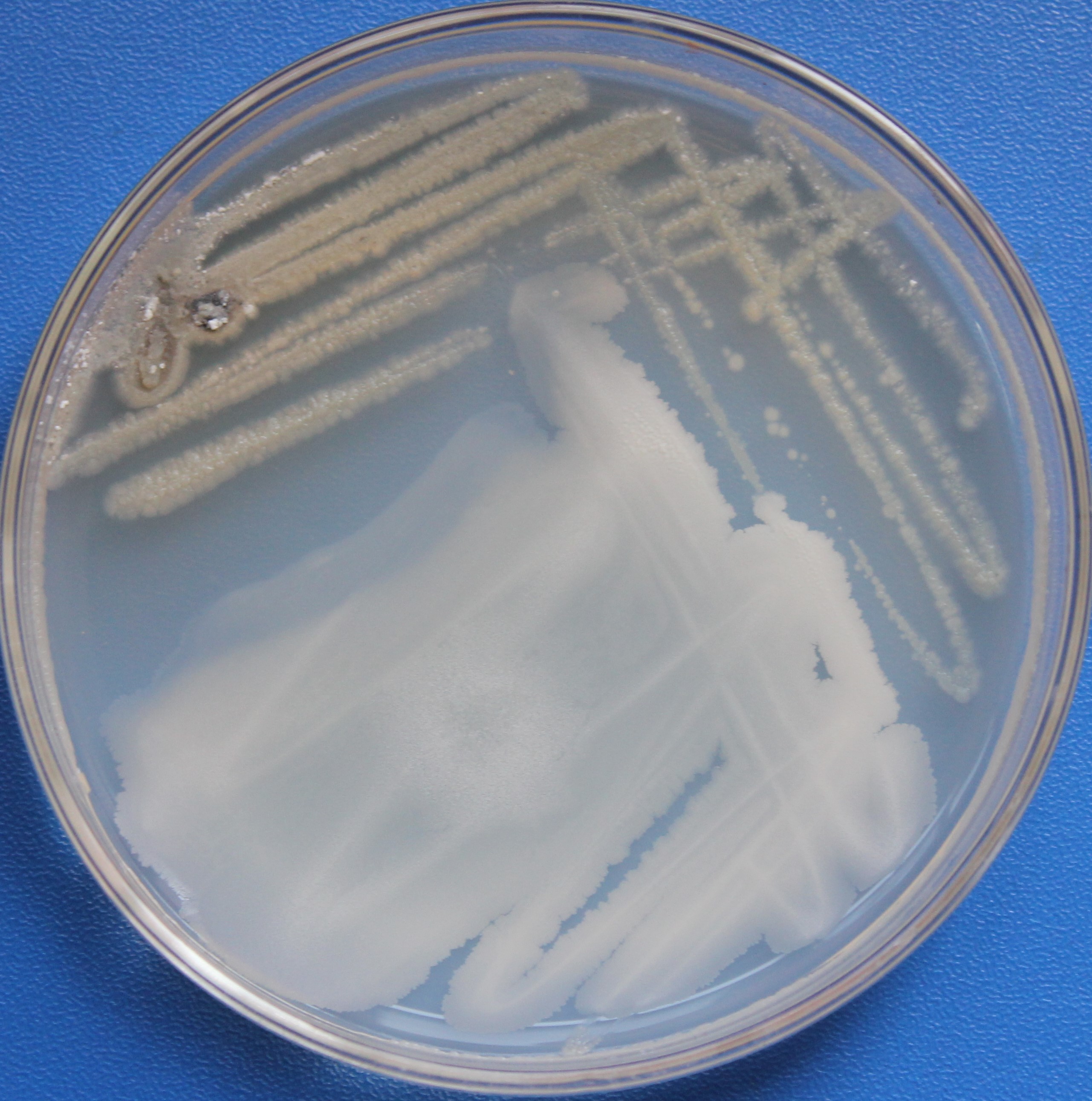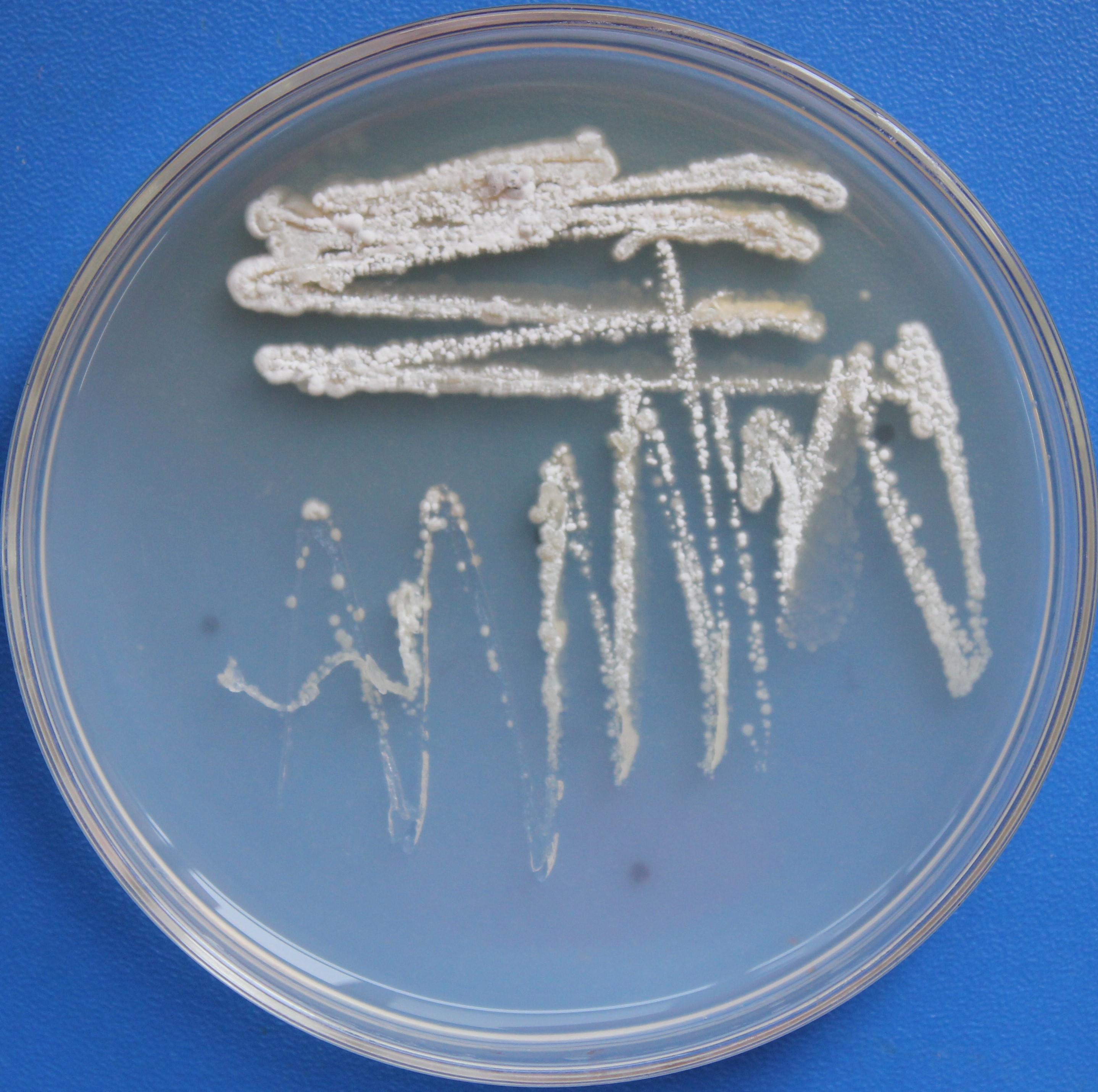 |
| --- | --- | --- | --- | --- | --- |
| ISP2 | Gost white | Silver | None | + |  |
| ISP3 | Snow | Light yellow | None | ++ |  |
| ISP4 | White | Gainsboro | None | +++ |  |
| ISP5 | Linen | Light yellow | None | ++ |  |
| ISP6 | Mint cream | Wheat | None | ++ |  |
| ISP7 | Bisque | Burlywood | None | + |  |
| PDA | Azure | Light yellow | None | + |  |

+ good growth; ++ better growth; +++ best growth

**Table S3.** Physiological and biochemical characteristics of strain JBS5-6

| **Characteristics** | **Results** |
| --- | --- |
| Temperature range for growth (℃) | 28-45℃ |
| pH range for growth | 5-9 |
| NaCl tolerance for growth (%) | 0-3 |
| Hippurate | + |
| H_2_S production | - |
| Milk peptonization | - |
| Starch hydrolysis | - |
| Cellulose hydrolysis | - |
| Gelatin liquefaction | - |
| Nitrate reduction | + |
| Melanoid pigment | - |
| Tyrosinase production | - |

+, Positive reaction; −, Negative reaction

**Table S4.** Carbon and nitrogen utilization characteristics of strain JBS5-6

| **Nitrogen Source Utiliziton** | | **Carbon Source Utilizition** | |
| --- | --- | --- | --- |
| Nitrogen Source | Result | Carbon Source | Result |
| L-arginine | - | α-Lactose | + |
| L-serine | + | D-Cellobiose | + |
| L-pheny | - | D-Fructose | + |
| Glycine | + | D-Galactose | + |
| Methionine | - | D-Glucose | + |
| Tryptophan | - | D-Mannose | + |
| L(+)-cysteine | - | D-Sorbitol | + |
| Phenylalanine | - | D-Trehalose | + |
| Valine | - | D-Xylose | + |
| Histidine | + | L-Arabinose | - |
| Glutamate | - | Melitose | - |
|  |  | Melibiose | + |
|  |  | D-Mannitol | + |
|  |  | Inositol | + |
|  |  | Melezitose | + |
|  |  | Rhamnose | + |
|  |  | Ribose | + |
|  |  | Maltose | + |
|  |  | Sucrose | + |
|  |  | sodium propionate | - |

+, Positive reaction; −, Negative reaction

**Table S5** Inhibitory activities of strain JBS5-6 extracts on [mycelial](../../../../D:/360%25E6%259D%2580%25E6%25AF%2592/Youdao/Dict/8.9.2.0/resultui/html/index.html#/javascript:;) [growth](../../../../D:/360%25E6%259D%2580%25E6%25AF%2592/Youdao/Dict/8.9.2.0/resultui/html/index.html#/javascript:;) and spore germination of the tested phytopathogenic fungi.

| **Pathogenic fungi** | **Mycelial Inhibition (%)** | **EC_50_ Value (µg/mL)** | **Inhibition of spore germination** | | |
| --- | --- | --- | --- | --- | --- |
|  |  |  | **1/2 × EC_50_** | **EC_50_** | **2 × EC_50_** |
| Foc TR4 (ATCC 76255) | 79.63 ± 1.79 ab | 136.92 ± 1.91 f | 45.82 ± 0.95 a | 69.84 ± 1.84 ab | 80.81 ± 0.99 b |
| *C. acutatum* (ATCC 56815) | 75.74 ± 1.16 c | 143.81 ± 1.68 de | 46.16 ± 1.67 a | 71.76±1.01 a | 83.25 ± 0.59 a |
| *C. fallax* (ATCC 38579) | 70.56 ± 1.47 de | 146.89 ± 0.83 c | 40.09 ± 1.56 cd | 62.15±0.94 d | 71.85 ± 0.98 d |
| *F. oxysporum. sp. cucumebrium* (ACCC 30220) | 65.93 ± 0.64 fg | 145.13 ± 3.02 cd | 37.01 ± 1.42 ef | 57.75 ± 0.53 f | 66.80 ± 1.24 f |
| *P. oryae* (ATCC 52352) | 63.15 ± 1.40 g | 142.15 ± 1.85 e | 35.58 ± 0.55 f | 55.58 ± 1.32 g | 64.31 ± 0.98 g |
| *C. gloeosporioides* (ATCC 58222) | 80.93 ± 0.85 a | 106.64 ± 1.82 h | 45.55 ± 1.59 a | 70.86 ± 1.06 a | 82.23 ± 0.96 ab |
| *F. graminearum* (ATCC 46779) | 77.59 ± 3.39 bc | 141.94 ± 2.76 e | 43.25 ± 1.35 b | 68.15 ± 0.96 b | 78.68 ± 0.71 c |
| *B. dothidea* (ATCC 208829) | 64.63 ± 2.25 g | 165.9 ± 1.78 a | 36.13 ± 0.95 ef | 56.12 ± 0.63 fg | 65.13 ± 0.38 g |
| *C. lunata* (ATCC 42011) | 81.48 ± 0.85 a | 108.96 ± 0.89 h | 45.53 ± 0.62 a | 71.16±0.89 a | 82.38 ± 1.15 ab |
| *C. fragariae* (ATCC 58718) | 68.15 ± 0.32 ef | 162.34 ± 1.60 b | 38.19 ± 0.98 de | 60.04 ± 1.03 e | 68.85 ± 0.89 e |
| *B. cinereal* (ATCC 11542) | 72.22 ± 0.56 d | 129.27 ± 0.85 g | 41.71 ± 1.27 bc | 64.12 ± 0.84 c | 71.48 ± 0.53 d |

Data in the table are means ± SD. Different lower-case letters in the same column showed values that were signiﬁcantly different at the P < 0.05 level by Duncan’s new multiple range test

**Table S6** MIC values of strain JBS5-6 extracts against the tested phytopathogenic fungi

| **Pathogenic fungi** | **MIC of 5-6**  **(µg/mL)** | **MIC of Cy**  **(µg/mL)** | **MIC of Be**  **(µg/mL)** |
| --- | --- | --- | --- |
| Foc TR4 (ATCC 76255) | ＞6.25 | ＞12.5 | ＞25.0 |
| *C. acutatum* (ATCC 56815) | ＞3.125 | ＞3.125 | ＞12.5 |
| *C. fallax* (ATCC 38579) | ＞12.5 | ＞3.125 | ＞3.125 |
| *F.oxysporum.sp.cucumebrium* (ACCC 30220) | ＞12.5 | ＞6.25 | ＞6.25 |
| *P. oryae* (ATCC 52352) | ＞50.0 | ＞12.5 | ＞25.0 |
| *C. gloeosporioides* (ATCC 58222) | ＞1.563 | ＞3.125 | ＞3.125 |
| *F. graminearum* (ATCC 46779) | ＞3.125 | ＞6.25 | ＞6.25 |
| *B. dothidea* (ATCC 208829) | ＞25.0 | ＞12.5 | ＞6.25 |
| *C. lunata* (ATCC 42011) | ＞3.125 | ＞1.563 | ＞3.125 |
| *C. fragariae* (ATCC 58718) | ＞12.5 | ＞6.25 | ＞12.5 |
| *B. cinerea* (ATCC 11542) | ＞12.5 | ＞12.5 | ＞6.25 |

Cy, Cycloheximide (antifungal agent); Az, Azoxystrobin (antifungal agent).

**Table S7.** Summary of strain JBS5-6 genome

| **Attribute** | **Value** | **% of total^a^** |
| --- | --- | --- |
| Genome size (bp) | 11161721 | 100 |
| DNA coding region(bp) | 9378117 | 84.02 |
| DNA G+C content(bp) | 7969468 | 71.40 |
| Total genes | 9840 | 100 |
| RNA genes^b^ | 73 | 0.74 |
| Protein-coding genes | 9767 | 31.24 |
| Genes assigned to COGs | 7213 | 73.85 |
| Genes assigned to GO | 6537 | 66.93 |
| Genes assigned to KEGG | 4432 | 45.38 |
| CRISPR repeat | 138 | 1.40 |

^a^The total is based on either the size of the genome in base pairs or the total number of proteins encoding genes in the annotated genome. ^b^RNA genes also include three rRNAs and 70 tRNA.

Table S8. Cluster number and gene number shown in different cluster types

| **Cluster Type** | **Cluster Number** | **Gene Number** |
| --- | --- | --- |
| Arylpolyene-ladder | 2 | 101 |
| Bacteriocin | 3 | 68 |
| Butyrolactone | 2 | 24 |
| Ectoine | 1 | 10 |
| hserlactone | 1 | 19 |
| indole | 1 | 22 |
| lantipeptide | 6 | 205 |
| NRPS | 10 | 310 |
| oligosaccharide | 1 | 52 |
| other | 3 | 93 |
| siderophore | 3 | 31 |
| PKS I | 23 | 373 |
| PKS II | 1 | 39 |
| PKS III | 2 | 72 |
| terpene | 6 | 120 |
| **Total** | **65** | **1539** |

**Table S9. Prediction and functional annotation of** [**secondary**](javascript:;) [**metabolite**](javascript:;)**s**

| **Region** | **Type** | **Gene position (bp)** | **Predicted compounds** | **Similarity** | **Functional annotation** | **References** |
| --- | --- | --- | --- | --- | --- | --- |
| Region 3 | NRPS, lassopeptide | 3710-63108 | Coelichelin | 90% | Peptide siderophore | Challis et al., 2000 |
| Region 3 | PKS I | 393743-426079 | Azalomycin F3a | 73% | Antimicrobial and anticancer activities | Yuan et al., 2013 |
| Region 4 | Terpene | 53949-75049 | Pristinol | 100% | Substrate binding | Klapschinski et al., 2016 |
| Region 17 | Terpene | 101335-123659 | Geosmin | 100% | Source of the earthy-musty smell | Liato et al., 2017 |
| Region 22 | PKS I NRPS-like | 134397-179068 | Amipurimycin | 90% | Antimicrobial and antiviral activities | Romo et al., 2019 |
| Region 30 | Terpene | 188320-209480 | 2-methylisoborneol | 100% | Source of the earthy-musty smell | Chou et al. 2017 |
| Region 30 | NRPS | 216874-275884 | Rhizomide A-C | 100% | Cytotoxic | Zhang et al., 2019 |
| Region41 | PKS I | 66-531 | Curamycin | 100% | Antibiotic | Gros et al., 1968 |
| Region42 | PKS I | 59-618 | halstoctacosanolide A | 77% | Antimicrobial activity | Tohyama et al., 2004 |
| Region43 | NRPS, PKS I | 51-743 | Myxothiazol | 71% | Antibiotic inhibiting the cytochrome b-c1 | Thierbach et al., 1981 |
| Region 44 | PKS I | 86562-159077 | Spore pigment | 83% | Spore pigment | Lee et al., 2005 |
| Region 55 | NRPS | 56234-88768;1-1147 | Echoside A-E | 100% | Inhibitory activities against DNA topoisomerase l and II | Zhu et al., 2014 |
| Region 80 | Ectoine | 23777-34181 | Ectoine | 100% | Osmotic pressure compatible solute | Gao et al., 2013 |
| Region 144 | Siderophore | 1-11026 | Desferrioxamine E | 100% | Chelating agent | Borgnapignatti et al., 2004 |
| Region 144 | Siderophore | 1-11026 | Desferrioxamine B | 80% | Removing excess iron | Codd et al., 2017 |
| Region 179 | PKS I | 1-16620 | Nigericin | 83% | Anticancer and inhibitor of vaccinia virus | Myskiw et al., 2010 |
| Region316 | NRPS | 49-327 | Xenotetrapeptide | 100% | Not reported | Kegler et al., 2014 |
| Region316 | NRPS | 49-347 | Bicornutin A1，A2 | 100% | Antibiotic | Böszörményi et al., 2009 |
| Region316 | NRPS | 46-311 | Luminmide | 100% | Cytotoxic compounds | Weyler et al., 2016 |
| Region316 | NRPS | 46-311 | IcosalideA, B | 100% | Antimicrobial activities | Dose et al., 2018 |

References

Borgnapignatti, C., Rugolotto, S., De Stefano, P., Zhao, H., Cappellini, M. D., Del, V. G. C., et al. (2004). Survival and complications in patients with thalassemia major treated with transfusion and deferoxamine. *Exp. Hematol.* 89(10), 1187-1193. doi:10.1016/j.exphem.2004.07.013

Böszörményi, E., Ersek, T., Fodor, A., Fodor, A. M., Földes, L. S., Hevesi, M., et al. (2009). Isolation and activity of *Xenorhabdus* antimicrobial compounds against the plant pathogens *Erwinia amylovora* and *Phytophthora nicotianae*. *Appl Microbiol. Sep.* 107(3), 746-59. doi:10.1111/j.1365-2672.2009.04249.x

Challis, G. L., Ravel, J. (2000). Coelichelin a new peptide siderophore encoded by the *Streptomyces coelicolor* genome: structure prediction from the sequence of its non-ribosomal peptide synthetase. *FEMS Microbiology Letters*. 187(2), 111-114. doi:10.1111/j.1574-6968.2000.tb09145.x

Chou, W. K., Gould, C. A., Cane, D. E. (2017). Incubation of 2-methylisoborneol synthase with the intermediate analog 2-methylneryl diphosphate. *J. Antibiot.*70(5), 625-631. doi:10.1038/ja.2017.24

Codd, R., Richardson, S. T., Telfer, T. J., Gotsbacher, M. P. (2018). Advances in the chemical biology of desferrioxamine b (dfob). *ACS Chem. Biol*. 13(1), 11-25. doi:10.1021/acschembio.7b00851

Dose, B., Niehs, S. P., Scherlach, K., Florez, L. V., Kaltenpoth, M., Hertweck, C. (2018). Unexpected bacterial origin of the antibiotic icosalide: two-tailed depsipeptide assembly in multifarious *Burkholderia* symbionts. *ACS Chem. Biol.* 13(9), 2414-2420 doi:10.1021/acschembio.8b00600

Gao S., Zhang L., Li D., Liu S., and Li X. (2013). Comparison of ectoine synthesis regulation in secreting and non-secreting strains of halomonas. *Ann. Microbiol.* 64(3), 1357-1361. doi: 10.1007/s13213-013-0779-6

Gros, E.G., Deulofeu,V., Galmarini, O.L., Frydman, B. (1968). Curamycin. II. Structure of the hydrolysis products "curacin" and "D-curamicose". *Experientia.* 24(4), 323-324. doi:10.1007/BF02140795

Kegler, C., Nollmann, F. I., Ahrendt, T., Fleischhacker, F., Bode, E., Bode, H. B. (2014). Rapid determination of the amino acid configuration of Xenotetrapeptide. *Chem. Bio. Chem.* 15(6), 826-828. doi:10.1002/cbic.201300602

Klapschinski, T. A., Rabe, P, Dickschat, J. S. (2016). Pristinol, a sesquiterpene alcohol with an unusual skeleton from *Streptomyces pristinaespiralis*. *Angew. Chem. Int. Ed. Engl.* 55(34), 10141-4. doi:10.1002/anie.201605425

Lee, T. S., Khosla, C., Tang, Y. (2005). Orthogonal protein interactions in spore pigment producing and antibiotic producing polyketide synthases. *J. Antibiot.* 58(10), 663-666. doi:10.1038/ja.2005.91

Liato, V, Aïder, M. (2007). Geosmin as a source of the earthy-musty smell in fruits, vegetables and water: Origins, impact on foods and water, and review of the removing techniques. *Chemosphere.* 181,9-18. doi:10.1016/j.chemosphere.2017.04.039

Myskiw, C., Piper, J., Huzarewich, R., Booth, T. F., Cao, J., He, R. (2010). Nigericin is a potent inhibitor of the early stage of vaccinia virus replication. *Antivir. Res.* 88(3), 304-310. doi:10.1016/j.antiviral.2010.10.001

Romo, A.J., Shiraishi, T., Ikeuchi, H., Lin, G. M., Liu, H. W. (2019). The amipurimycin and miharamycin biosynthetic gene clusters: unraveling the origins of 2-aminopurinyl peptidyl nucleoside antibiotics. *J. Am. Chem. Soc.*141(36). doi:10.1021/jacs.9b03021

Thierbach, G., Reichenbach, H. (1981). Myxothiazol, a new inhibitor of the cytochrome b-c1 segment of the respiratory chain. *BBA-Bioenergetics.* 638(2), 282-289. doi:10.1016/0005-2728(81)90238-3

Tohyama, S, Eguchi, T, Dhakal, R. P., Akashi, T., Otsuka, M., Kakinuma, K. (2004). Genome-inspired search for new antibiotics. isolation and structure determination of new 28-membered polyketide macrolactones, halstoctacosanolides A and B, from *Streptomyces halstedii* HC34. *Tetrahedron.* 60: 3999-4005. doi:10.1016/j.tet.2004.03.027

Weyler, C., Heinzle, E. (2016). Synthesis of natural variants and synthetic derivatives of the cyclic nonribosomal peptide luminmide in permeabilized *E. coli* Nissle and product formation kinetics. *Appl. Microbiol. Biot.* 101(1), 131-138. doi:10.1007/s00253-016-7770-7

Yuan, G., Hong, K., Lin, H., She Z. Li, J. (2013). New Azalomycin F analogs from mangrove *Streptomyces* sp. 211726 with activity against microbes and cancer cells. *Mar. Drugs.* 11(3), 817-829, doi:10.3390/md11030817

Zhang, X, Hindra, Elliot, M. A. (2019). Unlocking the trove of metabolic treasures: activating silent biosynthetic gene clusters in bacteria and fungi. Curr. *Opin. Microbiol.* 51, 9-15. doi:10.1016/j.mib.2019.03.003

Zhu, J., Chen, W., Li, Y. Y., Deng, J. J., Zhu, D. Y., Duan, J. Liu, Y. (2014). Identification and catalytic characterization of a nonribosomal peptide synthetase-like (NRPS-like) enzyme involved in the biosynthesis of echosides from *Streptomyces* sp. LZ35. *Gene*. 10. 546(2), 352-358. doi:10.1016/j.gene.2014.05.053
